# Outer Membrane Vesicles of Actinobacillus pleuropneumoniae Exert Immunomodulatory Effects on Porcine Alveolar Macrophages

**DOI:** 10.1128/spectrum.01819-22

**Published:** 2022-08-30

**Authors:** Zhuang Zhu, Fabio Antenucci, Hanne Cecilie Winther-Larsen, Kerstin Skovgaard, Anders Miki Bojesen

**Affiliations:** a Department of Veterinary and Animal Sciences, Faculty of Health and Medical Sciences, University of Copenhagen, Frederiksberg C, Denmark; b Section of Pharmacology and Pharmaceutical Biosciences, Department of Pharmacy, University of Oslo, Oslo, Norway; c Department of Biotechnology and Biomedicine, Technical University of Denmark, Kongens Lyngby, Denmark; University of Florida

**Keywords:** outer membrane vesicles, *Actinobacillus pleuropneumoniae*, proteomics, porcine alveolar macrophages, innate immune response, host-pathogen interaction

## Abstract

Outer membrane vesicles (OMVs) are spontaneously released by Gram-negative bacteria, including Actinobacillus pleuropneumoniae, which causes contagious pleuropneumonia in pigs and leads to considerable economic losses in the swine industry worldwide. A. pleuropneumoniae OMVs have previously been demonstrated to contain Apx toxins and proteases, as well as antigenic proteins. Nevertheless, comprehensive characterizations of their contents and interactions with host immune cells have not been made. Understanding the protein compositions and immunomodulating ability of A. pleuropneumoniae OMVs could help illuminate their biological functions and facilitate the development of OMV-based applications. In the current investigation, we comprehensively characterized the proteome of native A. pleuropneumoniae OMVs. Moreover, we qualitatively and quantitatively compared the OMV proteomes of a wild-type strain and three mutant strains, in which relevant genes were disrupted to increase OMV production and/or produce OMVs devoid of superantigen PalA. Furthermore, the interaction between A. pleuropneumoniae OMVs and porcine alveolar macrophages was also characterized. Our results indicate that native OMVs spontaneously released by A. pleuropneumoniae MIDG2331 appeared to dampen the innate immune responses by porcine alveolar macrophages stimulated by either inactivated or live parent cells. The findings suggest that OMVs may play a role in manipulating the porcine defense during the initial phases of the A. pleuropneumoniae infection.

**IMPORTANCE** Owing to their built-in adjuvanticity and antigenicity, bacterial outer membrane vesicles (OMVs) are gaining increasing attention as potential vaccines for both human and animal use. OMVs released by Actinobacillus pleuropneumoniae, an important respiratory pathogen in pigs, have also been investigated for vaccine development. Our previous studies have shown that A. pleuropneumoniae secretes OMVs containing multiple immunogenic proteins. However, immunization of pigs with these vesicles was not able to relieve the pig lung lesions induced by the challenge with A. pleuropneumoniae, implying the elusive roles that A. pleuropneumoniae OMVs play in host-pathogen interaction. Here, we showed that A. pleuropneumoniae secretes OMVs whose yield and protein content can be altered by the deletion of the *nlpI* and *palA* genes. Furthermore, we demonstrate that A. pleuropneumoniae OMVs dampen the immune responses in porcine alveolar macrophages stimulated by A. pleuropneumoniae cells, suggesting a novel mechanism that A. pleuropneumoniae might use to evade host defense.

## INTRODUCTION

Actinobacillus pleuropneumoniae is the causative pathogen of porcine contagious pleuropneumonia, which has remained one of the most serious respiratory diseases in pigs for decades since its first discovery in 1957 (1). To date, 19 serovars and 2 biovars of A. pleuropneumoniae have been identified according to the antigenicity of capsular antigens and β-NAD dependence (2–4). ApxI to -IV exotoxins represent the most important virulence factors, resulting in devastating pulmonary lesions during an A. pleuropneumoniae infection (5). Apx toxins are encoded by *apxCABD* operons, typical for RTX toxins where the activator protein ApxC in conjunction with the acyl carrier protein (ACP) activates the protoxin ApxA by the addition of acyl groups (6, 7). ApxB and -D are necessary for the extracellular secretion of the toxins (8). All A. pleuropneumoniae serovars secrete one or two toxins of ApxI to -III in various combinations together with ApxIV, which is secreted only *in vivo* by all serovars (7). Despite considerable efforts invested in the prevention of this infectious disease, effective control of A. pleuropneumoniae infection is still challenged by antigenic diversity and serovars with varying geographical presence over time (9).

Outer membrane vesicles (OMVs) are nano-sized particles (20 to 300 nm in diameter) secreted by virtually all Gram-negative bacteria investigated to date (10). Originating from the bacterial outer membrane, OMVs distinctly mimic the outer membrane in terms of structure and composition. OMVs thus present a repertoire of membrane-associated antigens derived from their parent cell, including both pathogen-associated molecular patterns (PAMPs) and pathogen-specific antigens. Both are able of activating the host’s innate and adaptive immune responses (11). The immunostimulatory capacity renders OMVs promising and safe vaccine candidates targeting the bacteria from which they derive. In addition, OMVs have been demonstrated to induce adjuvant effects, acting synergistically with antigens in the vaccine formula (12). Moreover, the content of OMVs can easily be modified by genetic engineering of their parent bacteria, which makes them ideal vehicles for delivery of antigens of interest be they host derived or of exogenous origin (13–16). The research on OMV-based vaccines has been inspired by the success of Bexsero, which is an OMV-containing vaccine licensed for human clinical use against meningitis B (17). Nowadays, increasing numbers of studies have focused on the development of OMV-based vaccines against a broader spectrum of pathogens, including bacteria and viruses (18, 19).

A. pleuropneumoniae has been shown to produce OMVs containing Apx toxins and proteases (20). However, A. pleuropneumoniae OMVs’ composition and possible biological roles are still poorly characterized. We recently reported on the immunoproteomic profile of OMVs from the A. pleuropneumoniae MIDG2331 Δ*nlpI* strain, a genetically engineered hypervesiculating mutant, and investigated the OMVs’ vaccine potential to protect against A. pleuropneumoniae infection through a series of *in vitro* and *in vivo* studies (21–23). Our data indicated that OMVs derived from A. pleuropneumoniae were able to elicit high titers of specific IgG antibodies in pigs. However, this vaccine prototype failed to confer protection against heterologous challenge, suggesting that the interaction between A. pleuropneumoniae OMVs and the host immune cells may be more complex (23).

To better understand the importance of specific membrane-derived proteins and their biological roles in the A. pleuropneumoniae pathogenesis, we performed label-free quantitative mass spectrometry to systematically characterize the proteome of OMVs from A. pleuropneumoniae. The wild-type strain MIDG2331 (here referred to as WT) and three additional mutants including a hypervesiculating Δ*nlpI* mutant, a Δ*palA* mutant devoid of the superantigen PalA, and the Δ*nlpI* Δ*palA* double-deletion mutant were chosen in this study. Further, the impact of OMVs on porcine alveolar macrophages (PAMs) was investigated. For the first time, we demonstrated that A. pleuropneumoniae OMVs can inhibit immune responses in PAMs stimulated by either inactivated or live bacteria.

## RESULTS

### Deletion of *nlpI* and *palA* leads to an aberrant cellular phenotype.

**FIG 1:**
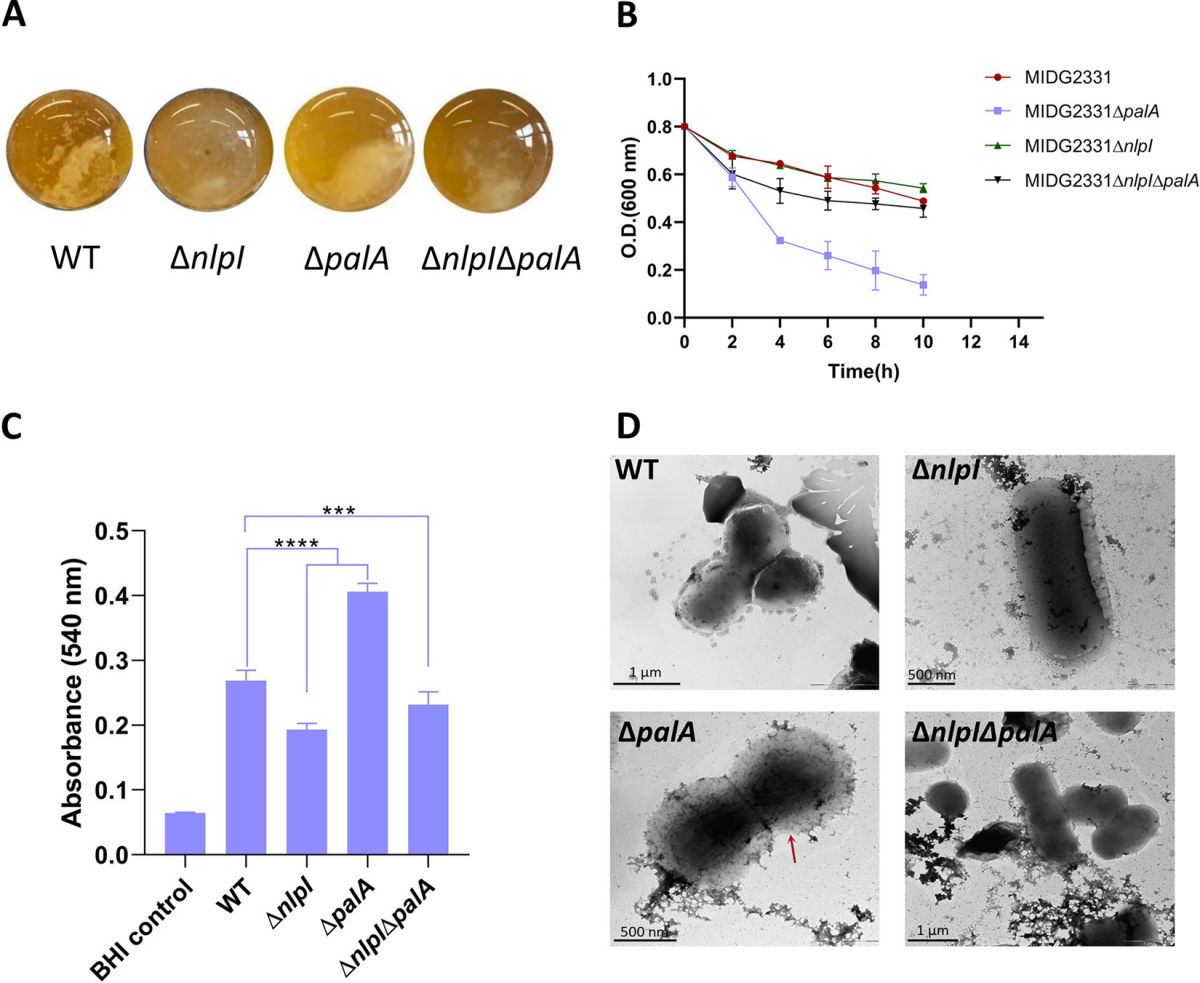
Deletions of *nlpI* and *palA* lead to an aberrant cellular phenotype. (A) A. pleuropneumoniae cells autoaggregate and settle down to the bottom of the tubes. (B) The autoaggregation of A. pleuropneumoniae was quantified by measuring the OD_600_ value of the upper bacterial suspensions every 2 h. Values are means ± standard deviations (SD) of absorbance calculated from biological duplicates. Statistically significant differences in areas under curves are found in the Δ*palA* mutant (*P* < 0.0001) and the Δ*nlpI* Δ*palA* mutant (*P* < 0.01) compared to WT. (C) Biofilm formation was quantified using the crystal violet staining method. The means ± SD from three experiments performed in duplicate are shown. Statistically significant differences compared to the WT cells are indicated with asterisks (***, *P* < 0.001; ****, *P* < 0.0001). (D) Transmission electron microscopy of A. pleuropneumoniae strains. The red arrow indicates a thick capsule-like structure found in the Δ*palA* mutant.

Both NlpI and Pal (peptidoglycan-associated lipoprotein) are well-known lipoproteins involved in maintaining the cell envelope integrity in Escherichia coli. To test whether they have similar biological functions in A. pleuropneumoniae, we examined changes of the cellular phenotype among WT and the three *ΔnlpI*, *Δpal*, and *ΔnlpI Δpal* mutants. First, we noticed that WT formed aggregates that settled down on the bottom of the culture tube, while the Δ*nlpI* mutant hardly formed aggregates (Fig. 1A). In contrast, the deletion of *palA* appeared to exacerbate the autoaggregation process compared with the WT strain, whereas the Δ*nlpI* Δ*palA* double-deletion mutant showed a phenotype intermediate between those of the Δ*nlpI* mutant and the Δ*palA* mutant (Fig. 1A). To quantitatively compare the formation of aggregates among the four strains, we performed an autoaggregation assay showing that the value of optical density at 600 nm (OD_600_) of the Δ*palA* mutant decreased faster than that of the other three strains (Fig. 1B), confirming that most aggregates were formed by the Δ*palA* mutant as observed in Fig. 1A. The areas under curves of the four strains were compared. Significant differences were found for both the Δ*palA* and Δ*nlpI* Δ*palA* mutants compared to the WT (Fig. 1B). These results indicate that the absence of PalA significantly enhances the autoaggregation behavior of A. pleuropneumoniae.

We also noticed that the Δ*palA* and Δ*nlpI* Δ*palA* strains had an increased ability to adhere to the culture tubes and form biofilms. Therefore, we hypothesized that their biofilm formation abilities were positively correlated with their autoaggregation ability, which is often regarded as the first step in biofilm formation (24). To investigate this hypothesis, a quantified biofilm formation assay was set up using the crystal violet staining method. The results obtained showed that the Δ*palA* mutant exhibited the strongest biofilm formation, followed by WT and the Δ*nlpI* Δ*palA* mutant, while the Δ*nlpI* mutant formed the least biofilm (Fig. 1C). It has been reported that PGA (poly-β-1,6-*N*-acetyl-d-glucosamine) is the major component of the biofilm matrix in A. pleuropneumoniae, which is synthesized by the *pga* operon and regulated by a two-component system, CpxAR. In addition, the dispersin B protein encoded by the *dspB* gene acts as a PGA-hydrolyzing enzyme dissolving the biofilm matrix to disperse cells (25, 26). We performed quantitative PCR (qPCR) to compare the expression levels of *pgaA*, *cpxA*, *cpxR*, and *dspB* genes in the three mutants. It appeared, however, that the expression levels of the four genes could not explain the different biofilm formation abilities. All three mutants had similar expression levels of these four genes, whether the biofilm-decreased Δ*nlpI* and Δ*nlpI* Δ*palA* mutants or the biofilm-increased Δ*palA* mutant (see Fig. S1 in the supplemental material), suggesting other factors govern the biofilm formation.

Transmission electron microscopy (TEM) imaging showed more cell aggregates for the WT and Δ*palA* and Δ*nlpI* Δ*palA* mutants but fewer for the Δ*nlpI* mutant (Fig. 1D), which is consistent with the results above. For the Δ*palA* mutant, the TEM imaging showed a large translucent area around the bacterial cell similar to a thick capsule-like structure that was absent in the other three strains.

### PalA and NlpI deficiency alter OMV production and size distribution.

**FIG 2:**
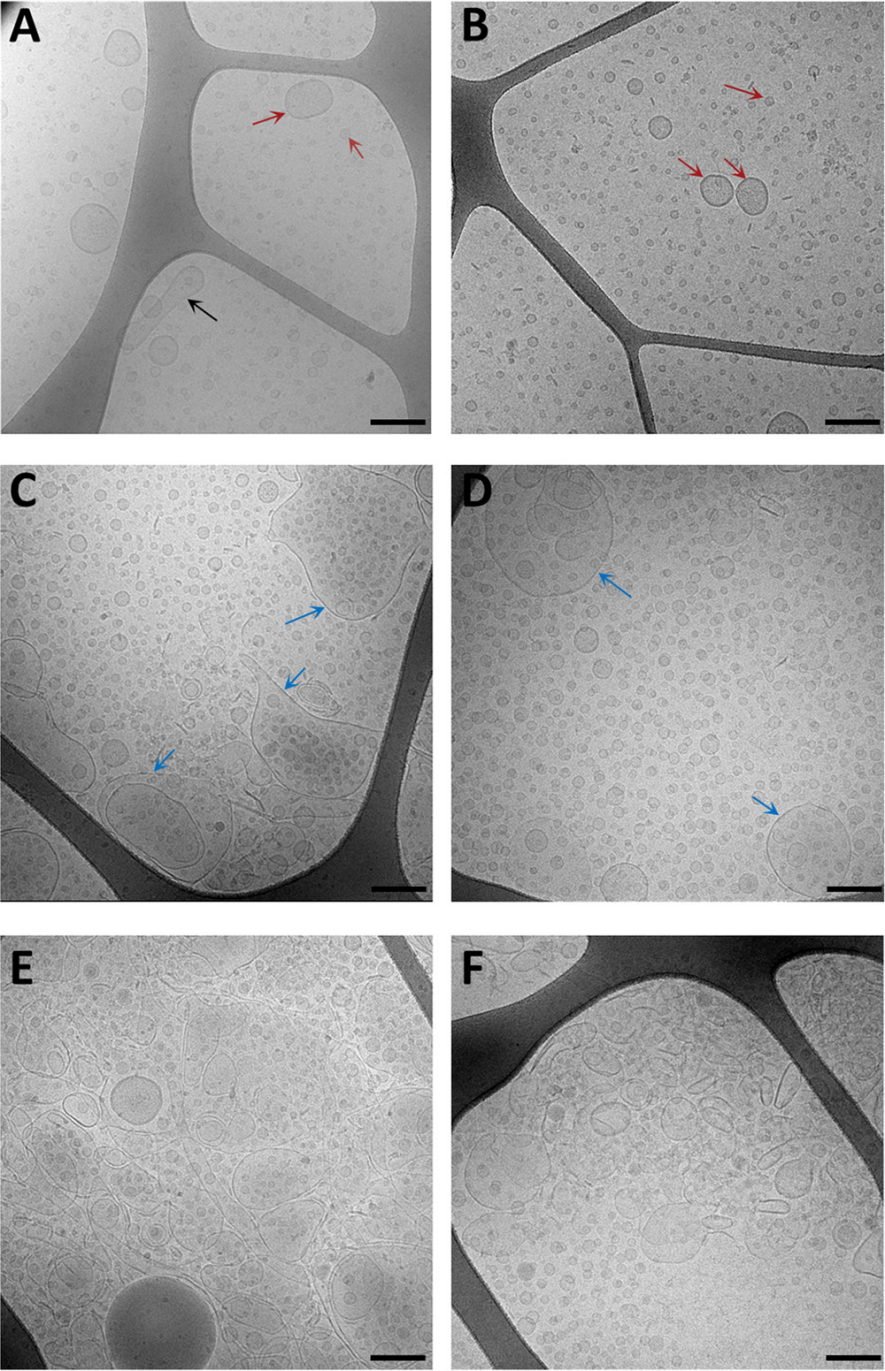
Cryo-TEM images of OMVs extracted from WT (A), Δ*nlpI* (B), Δ*palA* (C), and Δ*nlpI* Δ*palA* (D) strains. Typical sphere vesicles in the normal size range (20 to 200 nm) are indicated as red arrows. The black arrow indicates a long rod-shaped vesicle found in WT OMVs. Blue arrows indicate irregular and large membrane fragments found in Δ*palA* and Δ*nlpI* Δ*palA* mutants, which are more evident in graphs from Δ*palA* (E) and from Δ*nlpI* Δ*palA* (F) mutants. Bar, 200 nm.

OMVs from all four strains were extracted and visualized using cryo-TEM as shown in Fig. 2, in which OMVs from WT and Δ*nlpI* strains showed typical spherical structures with a regular size range from 20 to 200 nm (Fig. 2A and B). Besides, a few long rod-shaped vesicles were also observed in the WT (Fig. 2A). OMV extracts from the Δ*palA* and double-deletion mutants also contained considerable amounts of irregular membrane vesicles in diverse shapes and sizes in addition to the typical vesicles (Fig. 2C to F).

**FIG 3:**
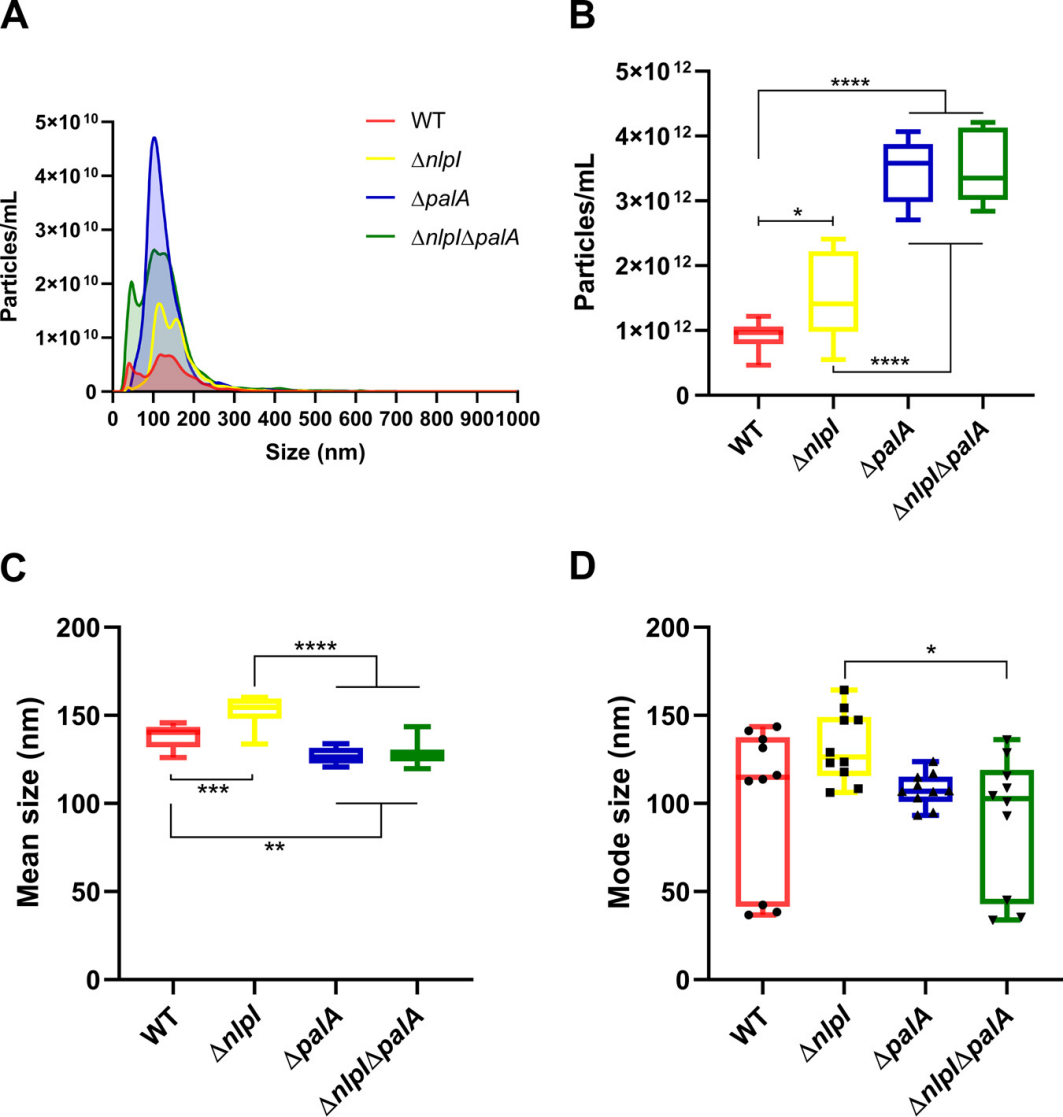
Size distribution and concentration of OMVs determined by nanoparticle tracking analysis. (A) The overall size distribution against corresponding concentration of OMVs from WT and three mutants. (B to D) The concentration (B), mean size (C), and mode size (D) of OMVs from WT and three mutants are shown as minimum to maximum values from biological duplicates with five technical repeats of each. Statistically significant differences among groups are indicated with asterisks (*, *P* < 0.05; **, *P* < 0.01; ***, *P* < 0.001; ****, *P* < 0.0001).

The size distribution and the concentration of the OMVs were determined by nanoparticle tracking analysis (NTA) as shown in Fig. 3A. The results indicated that deletion of *palA* and *nlpI*, respectively, increased the overall OMV production compared with WT (Fig. 2B). All mutants had an increased secretion of OMVs in the size range of 100 to 200 nm, which was particularly evident in the Δ*palA* mutant (Fig. 3A). Δ*nlpI* mutant OMVs had a significantly larger average size, while Δ*palA* and Δ*nlpI* Δ*palA* mutant OMVs had a significantly smaller average size than WT OMVs (Fig. 3C). The OMV size distribution curves of the WT, Δ*nlpI*, and Δ*nlpI* Δ*palA* strains had two peaks (Fig. 3A), corresponding to the OMVs of two groups of mode size where OMVs are most frequently detected from these three strains (Fig. 3D).

### *palA* deletion influences the protein profiles of A. pleuropneumoniae OMV extractions.

**FIG 4:**
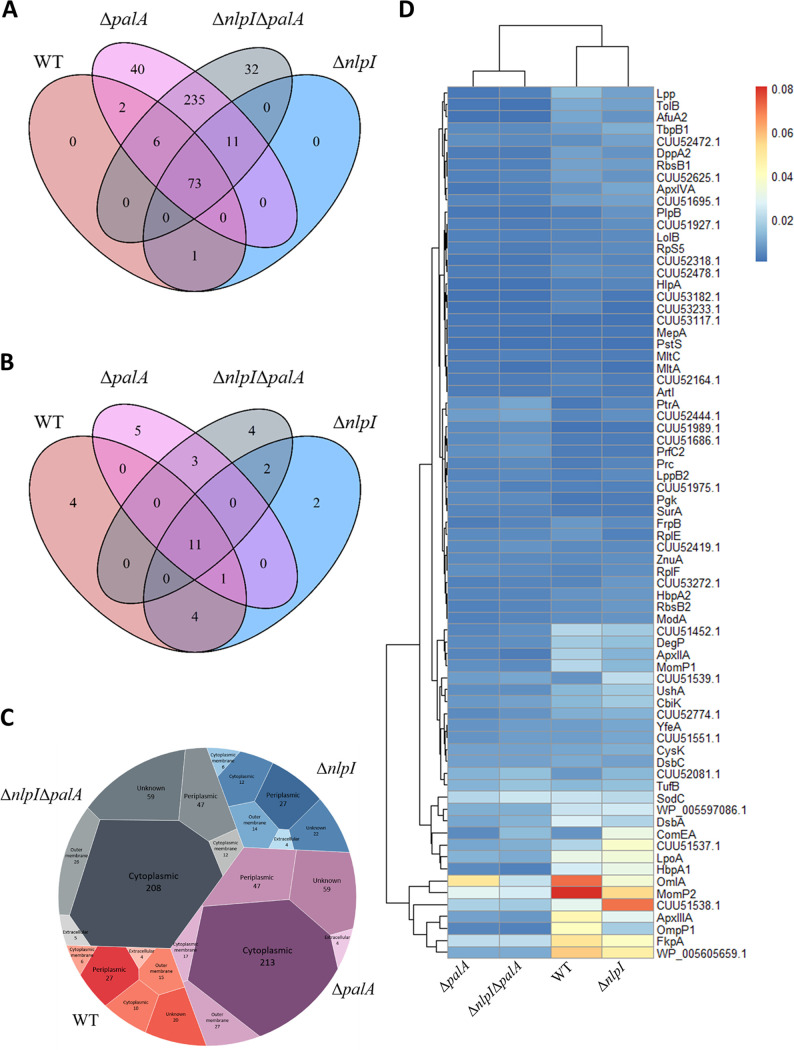
Proteomic analysis of OMVs from WT and three mutants. (A) Venn diagram showing shared and unique proteins identified in OMVs from WT and three mutants. (B) Shared and unique proteins among the top 20 most abundant proteins in OMVs from WT and three mutants are shown in the Venn diagram. (C) Subcellular localization analysis of proteins in OMVs is shown in the Voronoi diagram. OMVs from four different strains are represented by four blocks filled with different primary colors, which are further split according to the subcellular locations. The size and gradient colors reflect the number of proteins belonging to each subcellular localization. (D) The abundance of 73 shared proteins in OMVs from WT and three mutants is shown in the heat map, where the blue color represents low abundance and the red color represents high abundance determined by the values of normalized spectral abundance factor (NSAF). Proteins without available names are shown as accession numbers. Venn diagrams and the heat map were created with R Studio, and the Voronoi diagram was created with Tableau.

According to the morphological changes of the OMVs observed in the cryo-TEM images, we reasoned that the protein compositions of OMVs may have changed in the Δ*palA* and Δ*nlpI* Δ*palA* mutants, respectively. Therefore, a quantitative proteomic study was performed to compare the proteomes of OMVs from the A. pleuropneumoniae strains. A total of 82 and 85 proteins were detected in the OMVs from the WT and the Δ*nlpI* mutant, respectively, whereof 74 proteins were shared. Interestingly, the number of proteins detected in the Δ*palA* and Δ*nlpI* Δ*palA* mutant OMVs was 367 and 357, respectively, whereof 325 proteins were shared (Fig. 4A). The predicted subcellular localization of the additional proteins suggested most of the proteins from the Δ*palA* and Δ*nlpI* Δ*palA* mutant OMVs to be of cytoplasmic origin (Fig. 4C). We also compared the profiles of the top 20 most abundant proteins among four OMV extractions and showed that OMVs from the WT shared 16 out of the 20 most abundant proteins with the OMVs from the Δ*nlpI* mutant (Fig. 4B). However, OMVs from the WT shared only 12 and 11 out of the 20 most abundant proteins with those from the Δ*palA* and Δ*nlpI* Δ*palA* mutants, respectively (Fig. 4B).

The top 20 most abundant proteins of OMV extractions from each strain are listed in Table 1. The most abundant proteins in native OMVs spontaneously released by WT are mainly outer membrane and periplasmic proteins such as MomP2, OmlA, FkpA, OmpP1, LpoA, and extracellular toxin ApxIII. A considerable portion (9 out of 82) of WT OMV-associated proteins seem to be associated with iron uptake and transport, including HbpA1, TbpB1, FrpB, HbpA2, YfeA, AfuA2, FhuA, TbpA1, and a periplasmic protein of the ABC-type Fe^3+^-hydroxamate transport system (CUU53233.1). Gene ontology (GO) enrichment analysis revealed that WT OMV-associated proteins were significantly enriched in the biological processes of establishment of localization, transport, and ion transport (Fig. S2). In addition, cellular component analysis showed that outer membrane proteins and periplasmic proteins were highly enriched (Fig. S2). However, in the Δ*palA* and Δ*nlpI* Δ*palA* mutants, a high abundance of several ribosomal proteins such as RplV, RplQ, RpsP, RpsB, RplL, and RPlI was found (Table 1). A total of 73 proteins were present in the OMV extractions from all four strains (Fig. 4D). Interestingly, both ApxII and -III toxins were significantly less enriched in OMV extractions from Δ*palA* and Δ*nlpI* Δ*palA* mutants (Fig. 4D).

**TABLE 1:** Top 20 most abundant proteins identified in OMVs from A. pleuropneumoniae^*a*^

Abundance	WT	Δ*nlpI* mutant	Δ*palA* mutant	Δ*nlpI* Δ*palA* mutant
1	**MomP2**	**CUU51538.1**	**OmlA**	**MomP2**
2	**OmlA**	**MomP2**	**MomP2**	**SodC**
3	**WP_005605659.1**	**WP_005605659.1**	**FkpA**	**OmlA**
4	**FkpA**	**FkpA**	**SodC**	**FkpA**
5	ApxIIIA	**CUU51537.1**	**CUU51538.1**	**CUU51538.1**
6	OmpP1	**OmlA**	RplV	**TufB**
7	**LpoA**	**LpoA**	CUU52081.1	CUU52081.1
8	**CUU51538.1**	ComEA	**LpoA**	ComEA
9	**DsbA**	HbpA1	**TufB**	**DsbA**
10	HbpA1	ApxIIIA	**WP_005597086.1**	RplV
11	**WP_005597086.1**	**WP_005597086.1**	RplQ	**WP_005597086.1**
12	**SodC**	CUU51539.1	**WP_005605659.1**	**CUU51537.1**
13	MomP1	**SodC**	RpsP	**LpoA**
14	CUU51452.1	**DsbA**	**DsbA**	**WP_005605659.1**
15	**CUU51537.1**	OmpP1	**CUU51537.1**	RplL
16	ApxIIA	CUU51452.1	DsbC	RplI
17	DegP	CbiK	cysK	PtrA
18	Lpp	UshA	ApxIIIA	CUU52444.1
19	**TufB**	**TufB**	CUU52444.1	CUU51539.1
20	UshA	RpsJ	RpsB	CUU53419.1

a Proteins without available names are shown as corresponding accession numbers. Proteins in bold are common in the top 20 most abundant proteins of OMVs from four strains.

### A. pleuropneumoniae OMVs can be internalized by PAMs and are not cytotoxic.

**FIG 5:**
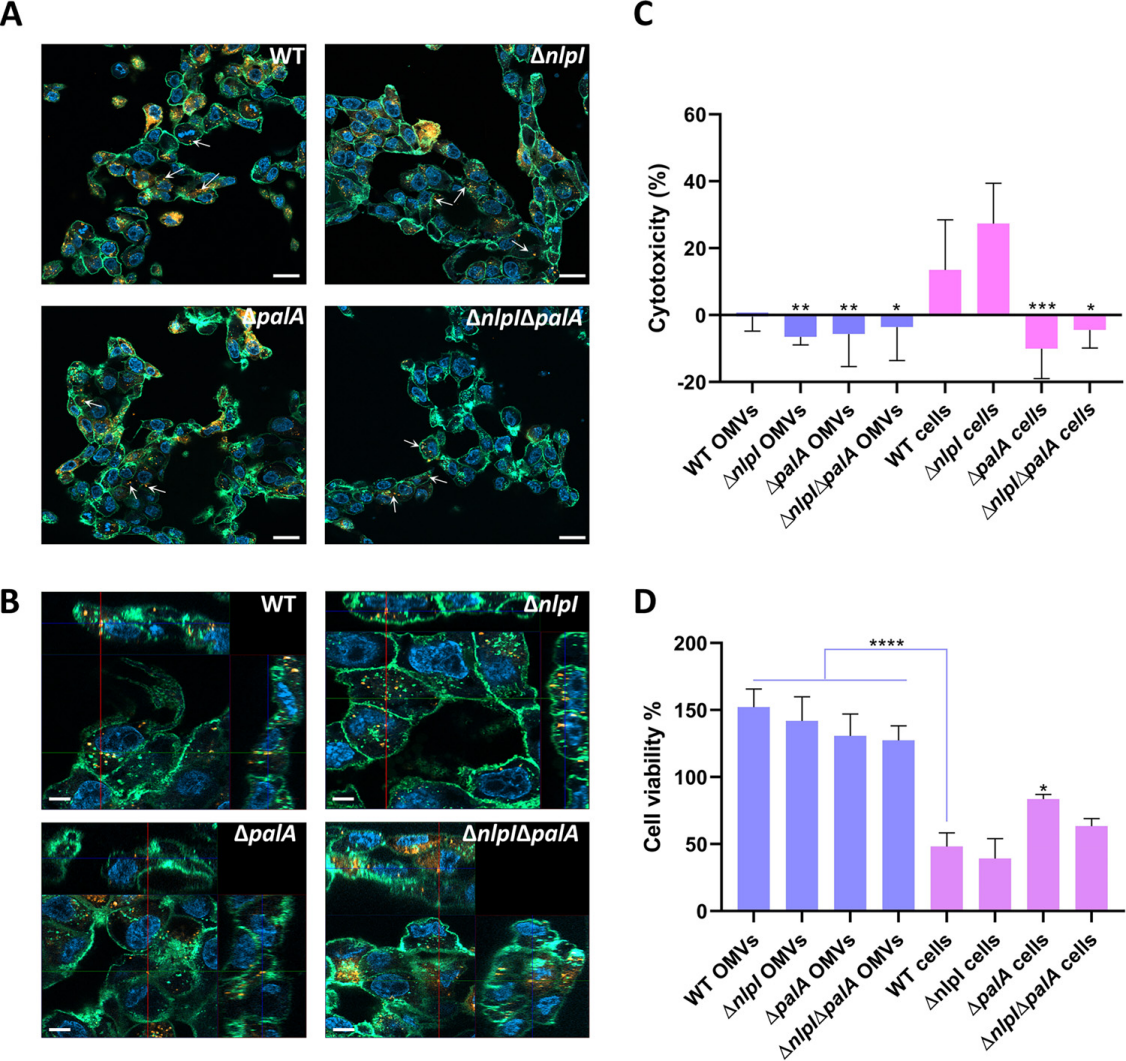
Internalization of OMVs into PAMs, as confirmed by confocal scanning laser microscopy. (A and B) Tiling images (5 × 5) (A) and Z-stack projections (B) are shown, where OMVs (indicated as arrows) are orange-red stained (DiI), cell membranes are green stained (fluorescein-conjugated wheat germ agglutinin), and cell nuclei are blue stained (DAPI). Bar, 20 μm and 5 μm in panels A and B, respectively. (C) The cytotoxicity of A. pleuropneumoniae OMVs and bacterial cells to PAMs was determined by measuring LDH released in the supernatant. (D) The viability of PAMs incubated with A. pleuropneumoniae OMVs and bacterial cells was determined using PrestoBlue reagent. Data represent means ± SD from three experiments performed in duplicate. Statistically significant differences from the WT cells are indicated with asterisks (*, *P* < 0.05; **, *P* < 0.01; ***, *P* < 0.001; ****, *P* < 0.0001).

To confirm if OMVs can be internalized by PAMs, confocal laser scanning microscopy was applied, results showed that most of the PAMs were able to internalize OMVs efficiently (Fig. 5A), and orthogonal images confirmed that the OMVs were localized inside the PAMs (Fig. 5B).

Due to the high abundance of ApxII and -III toxins detected in the OMVs, we hypothesized that the OMVs were cytotoxic to PAMs. However, none of the OMVs isolated from the WT or the mutants were able to induce a notable release of lactate dehydrogenase (LDH) between 6 and 24 h even at concentrations up to 20 μg/mL, suggesting no cytotoxic effect of any of the OMVs (Fig. S3). Based on these results, the highest concentration of 20 μg/mL OMVs and longest incubation time of 24 h were chosen for further investigation. Both the cytotoxicity and viability of PAMs were quantified after incubation with either OMVs or live bacteria at a multiplicity of infection (MOI) of 1:10. While both the WT and mutant bacteria induced a reduction in cell viability, the results showed that none of the OMVs from the four strains were cytotoxic to PAMs (Fig. 5C and D).

### A. pleuropneumoniae OMVs failed to induce significant expression of inflammatory and immune-related genes in PAMs.

All four strains and their corresponding OMVs were used to stimulate PAMs to compare the gene expression patterns. To gain first insights into the gene expression patterns of PAMs, a principal-component analysis (PCA) was carried out as shown in Fig. S4. All four OMV-stimulated groups clustered together and presented values similar to those of the phosphate-buffered saline (PBS)-treated control group and the lipopolysaccharide (LPS)-treated group, indicating only minor differences in gene expression patterns among these groups (Fig. S4). In contrast, some members of the groups stimulated by inactivated bacterial cells showed clearly distinct clusters, suggesting distinct gene expression patterns in groups stimulated by different A. pleuropneumoniae strains (Fig. S4).

**FIG 6:**
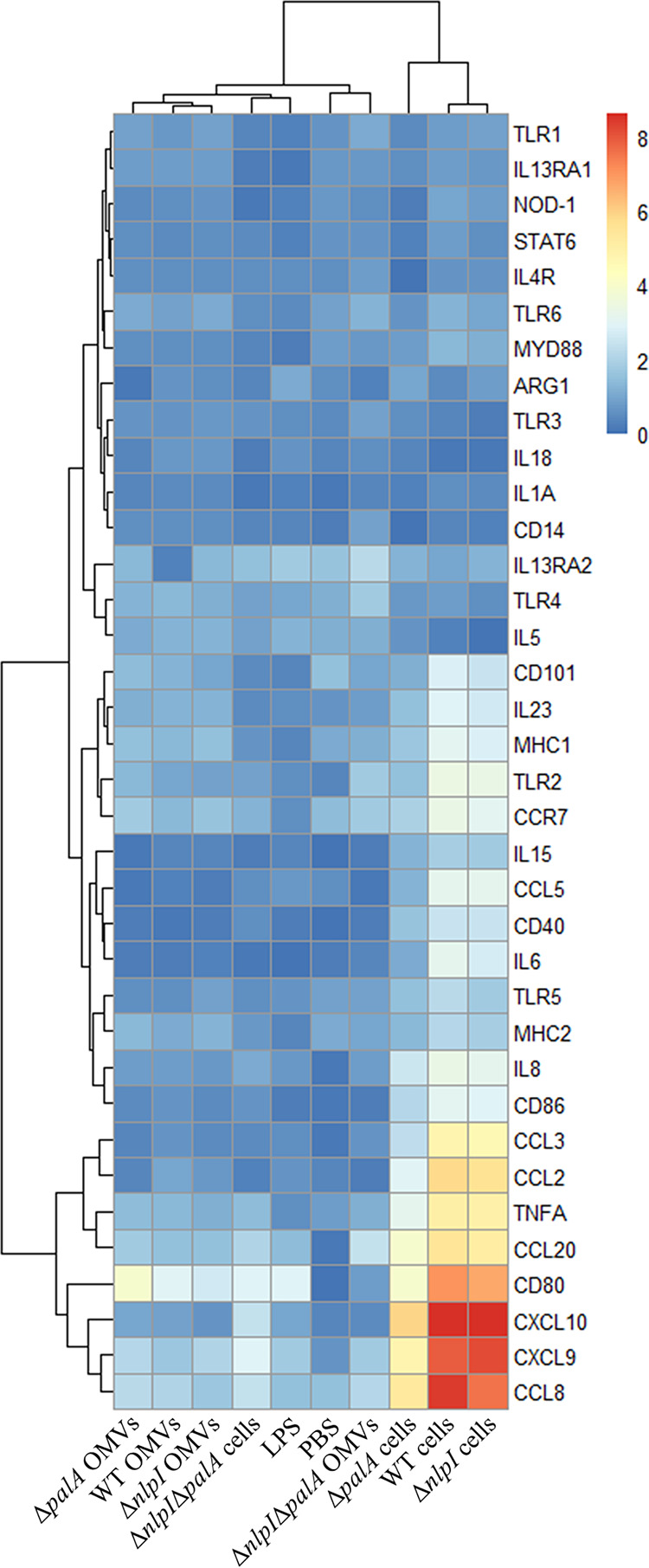
Heat map showing the log_2_ transformation of relative quantification (RQ) values of 36 immune-related genes in PAMs after 24-h stimulation. RQ values were calculated and scaled to the lowest value in each gene group after normalization of the *C_q_* values of 36 genes to the mean *C_q_* value of actin beta (ACTB), beta-2-microglobulin (B2M), and glyceraldehyde-3-phosphate (GAPDH). The blue color represents low expression level, and the red color represents high expression level. Created with R Studio.

A heat map integrated with two dendrograms was constructed to visualize the gene expression patterns in PAMs as shown in Fig. 6. As seen in the upper dendrogram, two distinct expression patterns represented by two main clusters were apparent, in which the cluster to the right represents high expression levels following treatment of WT cells, Δ*nlpI* cells, and Δ*palA* cells, while the cluster to the left represents low expression levels induced by all the OMV groups, PBS, LPS, and the Δ*nlpI* Δ*palA* mutant groups. Most of the genes that were highly upregulated (>20-fold increase) in the WT cluster are those for chemokines, including CXCL10, CXCL9, CCL8, CCL3, CCL2, CCL20, and costimulatory molecule CD80. Several other genes, including those for tumor necrosis factor (TNF), interleukin-8 (IL-8), IL-6, CCL5, CCR7, CD40, CD86, major histocompatibility complex class I (MHC-I), TLR2, TLR5, IL-15, and IL-23, were also upregulated, although to a lesser degree (>2-fold increase) in WT cell and Δ*nlpI* cell groups. KEGG analysis of the differentially expressed genes in the WT and Δ*nlpI* groups indicated a slight enrichment of inflammatory response-related pathways compared to all tested genes, including cytokine-cytokine receptor interaction, viral protein interaction with cytokine and cytokine receptor, Toll-like receptor signaling pathway, chemokine signaling pathway, TNF signaling pathway, and IL-17 signaling pathway, respectively, although no statistically significant association was found (Fig. S5).

### A. pleuropneumoniae OMVs inhibited the expression of cytokines in PAMs stimulated by inactivated or live A. pleuropneumoniae cells.

**FIG 7:**
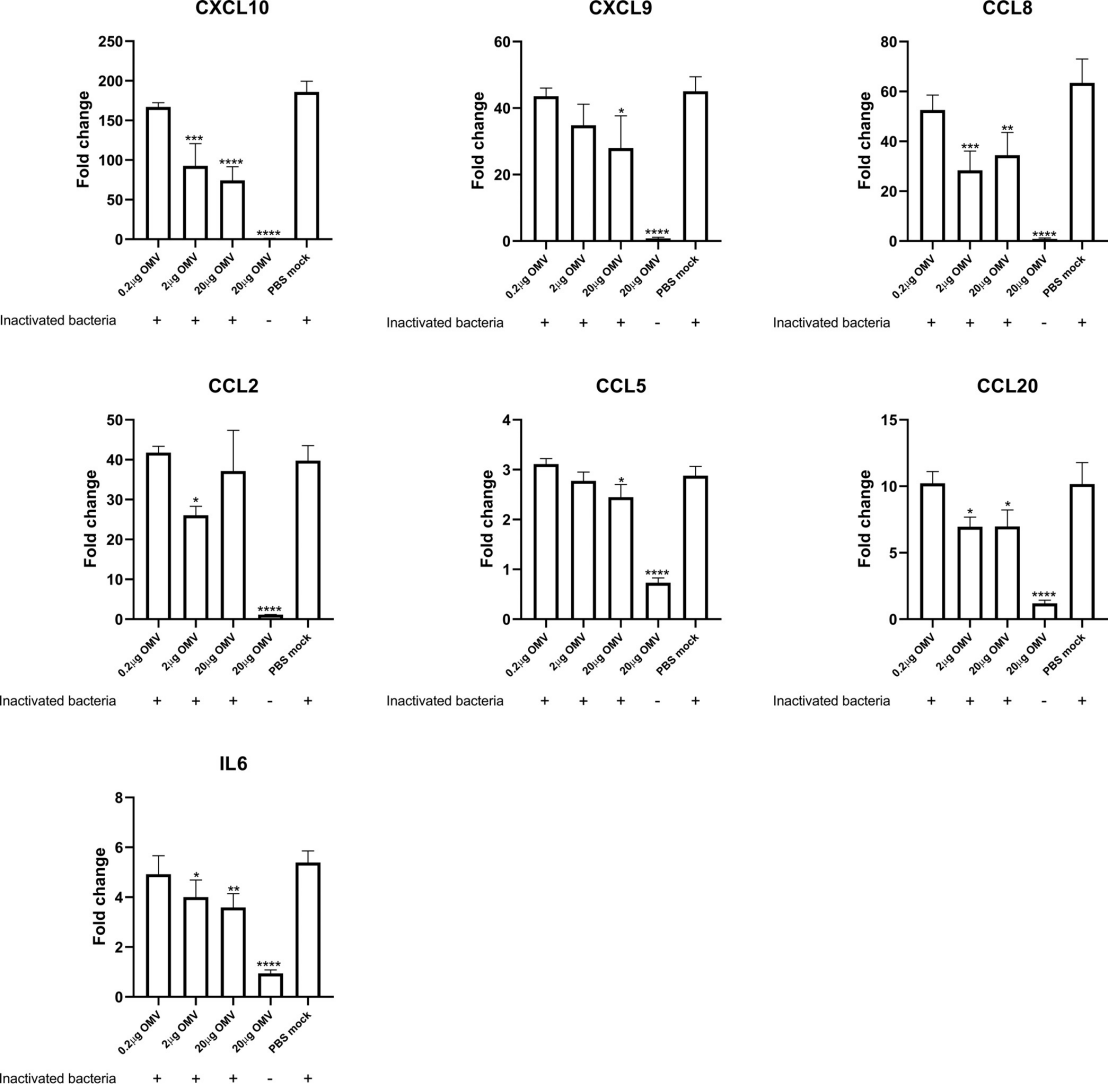
WT OMVs dampen immune responses of PAMs stimulated by inactivated A. pleuropneumoniae cells. Costimulation of PAMs with WT OMVs and inactivated WT cells for 24 h showed decreased expression of cytokines compared to PAMs stimulated with inactivated WT cells alone. Different doses of OMVs were applied to stimulate PAMs together with inactivated WT cells. Data represent means ± SD from biological triplicates. All fold change data are relative to the PBS-treated but unstimulated group. Statistically significant differences from the PBS-mock-treated group are indicated with asterisks (*, *P* < 0.05; **, *P* < 0.01; ***, *P* < 0.001; ****, *P* < 0.0001).

As mentioned above, A. pleuropneumoniae OMVs did not elicit a significant innate immune response in PAMs. On the contrary, it was observed that some of the genes investigated were downregulated by WT OMVs compared to the PBS-treated group, such as CCL5, IL-6, and TLR5, which made us wonder if the OMVs had the ability to inhibit, rather than stimulate, an innate immune response as observed in the case of whole cells of A. pleuropneumoniae. Therefore, the PAMs were stimulated with inactivated WT cells together with different doses of OMVs. Several genes that were significantly upregulated in the high-throughput qPCR analysis were chosen for further analysis, including CXCL10, CXCL9, CCL8, IL-8, CCL3, CCL20, CCL2, IL-6, CCL5, and TNF. As hypothesized, the upregulation observed for the genes following stimulation by inactivated bacterial cells was, for most genes, significantly inhibited by OMVs (Fig. 7). Interestingly, the inhibition seemed to take place at an OMV concentration as low as 2 μg/mL for CXCL10, CCL8, CCL20, and CCL2 (Fig. 7). Increasing the OMV concentration up to 20 μg/mL did not significantly enhance the inhibition, except for CXCL9 and CCL5, where significant inhibition was observed only at the concentration of 20 μg/mL OMVs (Fig. 7).

**FIG 8:**
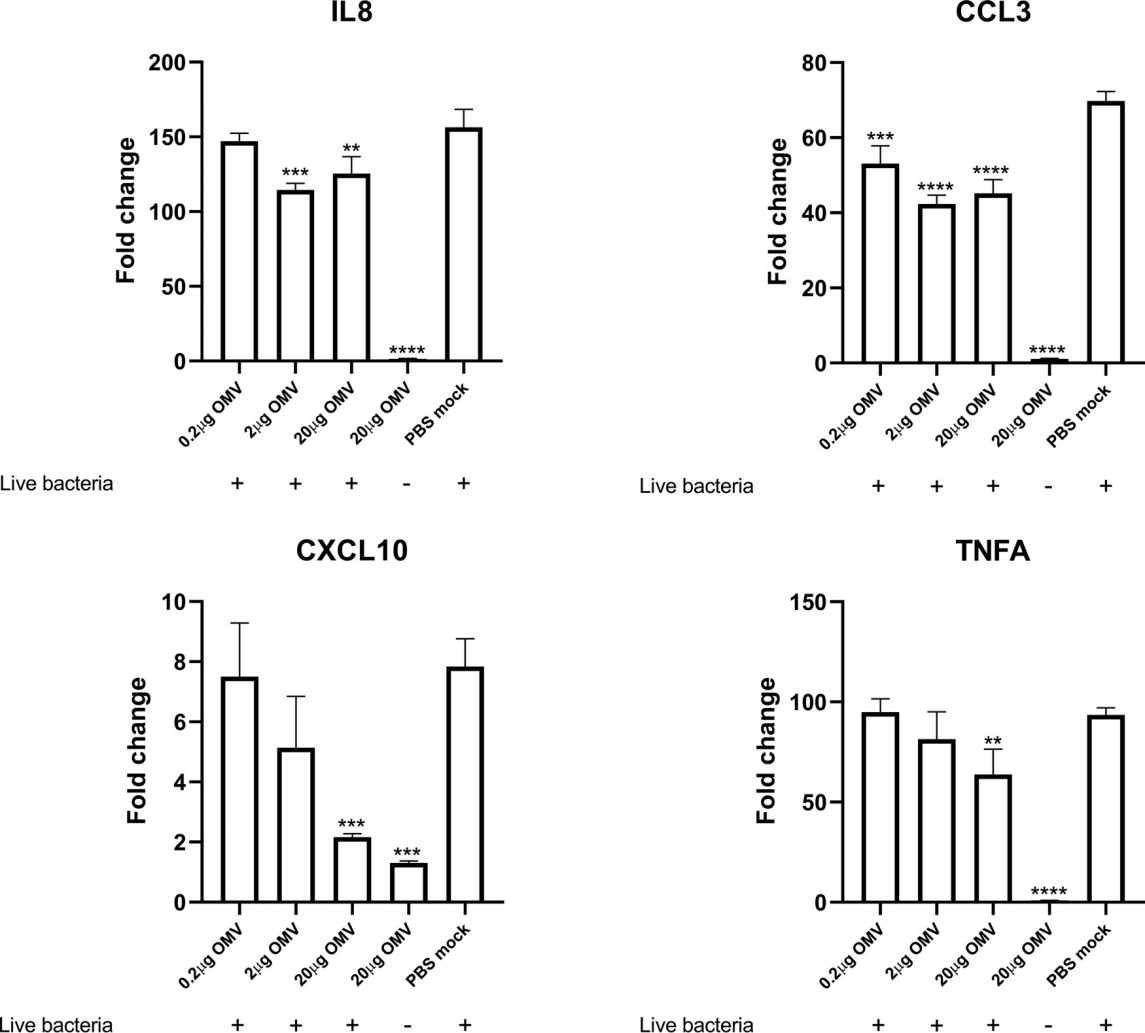
WT OMVs dampen immune responses of PAMs stimulated by live A. pleuropneumoniae cells. Costimulation of PAMs with WT OMVs and live WT cells for 24 h showed decreased expression of cytokines compared to PAMs stimulated with live WT cells alone. Different doses of OMVs were applied to stimulate PAMs together with live WT cells. Data represent means ± SD from biological triplicates. All fold change data are relative to the PBS-mock-treated but unstimulated group. Statistically significant differences from the PBS-mocked group are indicated with asterisks (*, *P* < 0.05; **, *P* < 0.01; ***, *P* < 0.001; ****, *P* < 0.0001).

Next, we wondered whether OMVs could inhibit the innate immune response of PAMs during a live A. pleuropneumoniae infection. PAMs were exposed to different doses of OMVs followed by A. pleuropneumoniae infection either after 24 h or simultaneously. The results showed that simultaneous stimulation of PAMs with live A. pleuropneumoniae and OMVs led to significantly reduced expression of IL-8, CCL3, CXCL10, and TNF compared with that of PAMs stimulated with A. pleuropneumoniae alone (Fig. 8). However, PAMs treated with OMVs for 24 h prior to A. pleuropneumoniae infection led to inhibition of only CXCL10 expression (Fig. S6).

## DISCUSSION

### Comparative proteomic analysis of A. pleuropneumoniae OMVs.

This study, for the first time, characterized a complete proteome of OMVs naturally released by A. pleuropneumoniae. The high enrichment of outer membrane proteins and periplasmic proteins found by subcellular localization prediction (Fig. 4C) and cellular component analysis (see Fig. S2 in the supplemental material) confirmed the outer membrane origin of WT OMVs and justified the method used for extracting OMVs. Consistent with our previously published results, we identified the presence and abundance of Apx toxins in OMVs (21). The parent strain MIDG2331 used in the current study was a serotype 8 clinical isolate, which secretes only ApxII and -III toxins *in vitro*. Both toxins were identified in our study, and the quantitative analysis showed that ApxIII toxin was the fifth most abundant protein in OMVs from the WT strain (Table 1). Intriguingly, ApxIV toxin, which is expected to be expressed *in vivo* only (27), was also identified in the OMVs, although in a much lower abundance than those of ApxII and -III toxins (Fig. 4D). As speculated in our previous study, the identification of ApxIV toxin may be due to the inability of the MASCOT algorithm employed at distinguishing between ApxII, ApxIII, and ApxIV degradation products (21). Also, proteins involved in iron uptake were observed to be enriched in WT OMVs, suggesting that OMVs may contribute to preserving and delivering iron to facilitate growth of A. pleuropneumoniae in the porcine respiratory tract, a highly iron-limited environment (28). Alternatively, packaging of these proteins may simply be due to their subcellular location in the outer membrane or periplasmic space. In our previous study, a hypervesiculating mutant was constructed by deleting the *nlpI* gene, whose absence has also been reported to induce higher OMV yields in other bacteria (29, 30). When comparing the proteomes of OMVs from the WT and Δ*nlpI* mutant, we found a high similarity between their protein profiles in terms of both composition and abundance. Approximately 90% of the proteins were shared among all proteins identified (74 out of 82 in WT and 74 out of 85 in the Δ*nlpI* mutant), as well as in the top 20 most abundant proteins (16 out of 20). The similar protein profiles of OMVs from the WT and the Δ*nlpI* mutant indicate that NlpI deficiency increased OMV production without compromising membrane integrity, which often leads to a leak of cytoplasmic proteins toward OMV extractions (31). The possible mechanism of NlpI modulating vesiculation has been proposed in E. coli, where the hypervesiculation phenotype was attributed to the increased peptidoglycan synthesis and decreased cross-links between the outer membrane and peptidoglycan layer (32). Interestingly, unlike in other bacteria where NlpI has been reported as an outer membrane lipoprotein (33), our analysis shows that NlpI in A. pleuropneumoniae has a predicted subcellular localization on the cytoplasmic membrane, which is supported by our finding that NlpI was absent in WT outer membrane vesicles while present in the Δ*palA* mutant (Data Set S1), where the membrane integrity seemed compromised. Our results support previous findings which suggested the outer membrane localization of NlpI in A. pleuropneumoniae, yet with low confidence due to lack of reproducibility in outer membrane preparations extracted using different protocols in the study (34).

Pal is a highly conserved outer membrane lipoprotein responsible for linking the outer membrane with the peptidoglycan layer in Gram-negative bacteria. From interaction with several other proteins, Pal is engaged in the Tol-Pal system, which is involved in maintaining outer membrane integrity. Deletion of Pal in E. coli showed increased outer membrane permeability, OMV formation, and sensitivity to vancomycin and sodium dodecyl sulfate (SDS), respectively, supporting the severe cell envelope defects observed in the mutant (35). Moreover, the defect induced by Pal depletion cannot be restored by overexpression of other proteins involved in outer membrane integrity like Lpp or TolA, while overexpression of Pal can complement the Lpp deletion, indicating an indispensable role of Pal for normal bacterial growth (35). However, the characterization of the physiological role of PalA in A. pleuropneumoniae has not been reported yet.

PalA has been suggested to be a superantigen that, at least partly, obscures protective immune responses otherwise elicited by immunization with Apx toxins (36). To verify the effect of the superantigen PalA on OMV composition and characteristics, we deleted the *palA* gene to create the Δ*palA* and Δ*nlpI* Δ*palA* mutants, respectively. Deletion of *palA* significantly increased the number of proteins identified in the OMVs secreted by the Δ*palA* mutant (367 proteins) and Δ*nlpI* Δ*palA* mutant (357 proteins) compared to the WT (82 proteins) and the Δ*nlpI* mutant (85 proteins). OMVs from the Δ*palA* mutant (325 out of 367 proteins) and the Δ*nlpI* Δ*palA* double mutant (325 out of 357 proteins) shared approximately 90% of the identified proteome, suggesting the comparable increase in protein content compared to WT and Δ*nlpI* OMVs can be exclusively attributed to the deletion of the *palA* gene. The subcellular localization analysis showed that most of the additional proteins were of cytoplasmic origin (Fig. 4C), which suggests PalA may have a membrane-preserving function. This suggestion was also supported by the presence of large amounts of membrane fragments (Fig. 2E and F) and the high abundance of ribosomal proteins (Table 1) observed only in OMVs from the PalA-deleted mutants, possibly as a result of compromised membrane integrity or even cell lysis. The membrane fragments found in OMVs from Δ*palA* and Δ*nlpI* Δ*palA* mutants appear similar to the consequence of cell explosive lysis (37), which could be a possible reason for the presence of membrane fragments and enhanced biofilm formation in Δ*palA* and Δ*nlpI* Δ*palA* mutants.

### Interaction between OMVs and porcine alveolar macrophage cell line.

In various species of Gram-negative bacteria, OMVs have been reported to be able to interact with host cells and exert different effects, including delivery of virulence factors and modulation of the immune response. To understand whether A. pleuropneumoniae OMVs exert similar properties, we characterized their immunomodulatory effect on a porcine alveolar macrophage cell line, which we regard as an integral part of the first line of defense in pigs during an A. pleuropneumoniae infection (38). First, we confirmed that A. pleuropneumoniae OMVs can be internalized by macrophages (Fig. 5A and B). Since Apx toxins, particularly the highly toxic ApxIII, were identified in OMVs, we hypothesized that A. pleuropneumoniae OMVs might be cytotoxic to PAMs. If so, OMVs might also act as vehicles delivering virulence factors into host cells, as reported for other bacteria (39–41). Surprisingly, our results showed that A. pleuropneumoniae OMVs did not exhibit the expected cytotoxicity on PAMs (Fig. 5C and D). A reasonable explanation for this observation could be that Apx toxins packed into OMVs require activation prior to exerting their cytotoxic effect, a theory which is supported by the liquid chromatography-mass spectrometry (LC-MS) results in this study showing that Apx toxins lack the acetylation required for the activation of Apx toxins (Fig. S7). Nonactivated Apx toxins originate from the cytoplasm, where they reside until concerted activation and secretion via the type I secretion system (6). Prior to activation and secretion, nonactivated Apx toxins may get access to the OMVs where type I secretion proteins are absent. Although previous studies have also identified Apx toxins in OMVs, it is still unknown whether these toxins are biologically functional. Our investigation provides evidence, at least indirectly, suggesting that Apx toxins carried by A. pleuropneumoniae OMVs are not functional.

The analysis of the immunostimulatory effect of OMVs on PAMs revealed that, surprisingly, none of the A. pleuropneumoniae OMVs from the WT or the three mutants appeared able to elicit an inflammatory stage in PAMs, whereas the inactivated parent cells were able to do so. The WT cells and Δ*nlpI* cells induced a similar expression profile in PAMs, while Δ*palA* cells induced a somewhat similar but dampened immune response (Fig. 6). The combined effect of deleting both *nlpI* and *palA* severely reduced the immunostimulatory ability of the Δ*nlpI* Δ*palA* cells (Fig. 6). These results support previous suggestions which characterized PalA as having important roles in the interaction between host immune cells and A. pleuropneumoniae. This was the case not only in A. pleuropneumoniae, where PalA was found to be a superantigen (36), but also in other bacterial species, where PalA has been found to be strongly immunogenic and immunodominant but not protective. For example, Pal in Bordetella pertussis was found to be an immunodominant lipoprotein eliciting strong serum antibody responses in mice. However, mice immunized with Pal alone were not protected against respiratory tract infection with B. pertussis (42). Valentine et al. also reported that neither polyclonal nor monoclonal IgG antibodies directed against Pal were able to protect mice from sepsis induced by E. coli (43). Immunoproteomic analysis showed that Pal in Actinobacillus actinomycetemcomitans is a highly immunoreactive lipoprotein, able to elicit considerable antibody production in rabbits (44). Likewise, Pal in Bordetella pertussis was found to be one of the proteins dominating the MHC-II-presented epitopes (45). Accordingly, these studies suggest that Pal in these species may act as a superantigen dominating the immune stimulation and thereby interfering with the immune response against other antigens. Under these circumstances, we believe that it would be necessary to remove this OMV-associated but nonprotective protein if OMVs were intended for vaccine development. Surprisingly, LPS, a potent agonist of the innate immune response, appeared not to induce significant immune responses in PAMs under the conditions tested. One possible explanation could be that PAMs are not as sensitive to LPS as other cell types, such as dendritic cells. In particular, purified free LPS should not be able to access the cytosol of macrophages and thereby activate the associated sensing pathway (46). Similar results were found in a study investigating OMVs produced by Haemophilus parasuis, a bacterial pathogen closely related to A. pleuropneumoniae, where LPS induced minor cytokine responses in primary PAMs compared to those induced by OMVs or bacterial cells (31).

Although the OMVs investigated in the current study failed to stimulate PAMs, our hypothesis stating that OMVs can inhibit PAM stimulation by A. pleuropneumoniae was confirmed (Fig. 7 and 8). This finding supports our previous suggestion that A. pleuropneumoniae OMVs may be involved in A. pleuropneumoniae pathogenesis (21), facilitating infection of A. pleuropneumoniae by impairing, at least partly, the host immune response. A similar phenomenon has also been reported in several other bacterial species, in which OMVs were found to dampen the immune response of the host cells through various mechanisms. For example, Pseudomonas aeruginosa OMVs have been demonstrated to reduce the secretion of IL-8 *in vitro* and *in vivo* by delivering interfering short RNAs targeting host mRNAs (47). Vibrio cholerae has been found to adopt a different strategy relying on OMV-based delivery of self-producing short RNAs into host intestinal epithelial cells, which in turn stimulate enhanced expression of microRNA capable of inhibiting the inflammatory response (48). Although we demonstrated that MIDG2331 OMVs repress the immune response of PAMs, OMVs from other serotypes of A. pleuropneumoniae would have to be investigated to confirm the OMV-mediated immunosuppression effect observed in this study, and the mechanism by which OMVs exert inhibitory effects still remains unknown. Future studies unraveling the underlying mechanism may help us understand the pathogenesis of A. pleuropneumoniae and the roles that OMVs play during infection.

Moreover, this study was limited to a single cell line, porcine alveolar macrophages, which means that different effects may be seen from interactions with other host cell types. In healthy individuals, alveolar macrophages are pivotal for maintaining lung immune homeostasis. They are responsible for clearing apoptotic cells, cellular debris, and other innocuous antigens without eliciting unnecessary immune responses that could harm the host. However, when the lung is infected, alveolar macrophages are able to rapidly respond and initiate a proinflammatory response (49). In humans, alveolar macrophages have been demonstrated to be poor at antigen presentation (50, 51), so although the OMVs in the current investigation failed to stimulate PAMs, more studies including additional cell types and possibly animal models are needed before conclusions on the immunostimulatory abilities of A. pleuropneumoniae OMVs are reached with certainty.

## MATERIALS AND METHODS

### Bacterial strains and growth conditions.

Bacterial strains and primers used to construct mutants are listed in Tables S1 and S2 in the supplemental material. To construct *ΔpalA* and Δ*nlpI* Δ*palA* mutants, upstream and downstream fragments of the *palA* gene as well as a chloramphenicol resistance cassette consisting of the chloramphenicol acetyltransferase gene and uptake signal sequence required for natural transformation in A. pleuropneumoniae were amplified using primers up-F/R, down-F/R, and Cm-F/R, respectively. These fragments as well as linearized vector pJET1.2/blunt (ThermoFisher Scientific) were ligated together in one step using the In-Fusion cloning kit (TaKaRa), thus giving rise to the plasmid pJET1.2-up-Cm-down, in which a chloramphenicol resistance cassette was flanked with upstream and downstream fragments of the *palA* gene. Mutants were constructed by homologous recombination using the plasmid pJET1.2-up-Cm-down naturally transformed into either the WT or Δ*nlpI* strain to produce *ΔpalA* and Δ*nlpI* Δ*palA* mutants as described previously (22, 52). All A. pleuropneumoniae strains were cultured in brain heart infusion (BHI) broth or on BHI agar supplemented with 0.01% NAD at 37°C. When necessary, the media were supplemented with 1.5 μg/mL chloramphenicol and/or 10 μg/mL trimethoprim.

### Characterization of A. pleuropneumoniae strains.

Autoaggregation was determined as previously described with minor modifications (53). Briefly, bacterial cells harvested from early stationary phase were washed three times and adjusted to an optical density value at 600 nm (OD_600_) of 0.8 in 10 mL phosphate-buffered saline (PBS). Tubes containing bacterial suspensions were allowed to stand statically at room temperature. Every 2 h, 1 mL of the upper bacterial suspensions was taken for measuring the OD_600_ value. Autoaggregation was determined by recording the decreasing OD_600_ values of upper bacterial suspensions due to the aggregated and settled bacterial cells.

To quantify the biofilm formation capacity, 100 μL of the A. pleuropneumoniae cultures at an OD_600_ value of 0.05 diluted from overnight cultures was aliquoted into sterile polystyrene 96-well microplates and incubated at 37°C for 24 h at 120 rpm. The liquid phase was then discarded, and the plate was washed twice with PBS followed by the addition of 125 μL of 0.1% crystal violet. After 30 min of incubation, the plate was washed three times and left to dry, and then 150 μL of 33% acetic acid was added to release the biofilm. Finally, 100 μL of the resulting suspension was transferred to a clean microplate for OD measurement at 540 nm (54).

To confirm if the autoaggregation and biofilm formation were associated with the expression level of biofilm-associated genes in A. pleuropneumoniae, RNA was extracted from bacterial cultures harvested in exponential phase using a FastPrep cell disrupter system (Qbiogene) and RNeasy minikit (Qiagen) incorporated with on-column DNase digestion to remove genomic DNA. Purified total RNA was quantified by a NanoDrop 1000 spectrophotometer (ThermoFisher Scientific) and reverse transcribed into cDNA using the High-Capacity RNA-to-cDNA kit (ThermoFisher Scientific). cDNA products were 10-fold diluted and used as the template for qPCR, which was performed using FastStart Essential DNA Green Master (Roche) on a LightCycler 96 instrument (Roche) under the following conditions: 95°C for 10 min followed by 45 cycles of 95°C for 10 s, 60°C for 10 s, and 72°C for 15 s. The relative expression level was normalized to the 16S rRNA gene and determined by the threshold cycle (2^−ΔΔ^*^CT^*) method (55). Primer sequences are listed in Table S3.

For transmission electron microscopy (TEM), A. pleuropneumoniae cells were harvested by centrifugation from the culture at an OD_600_ value of 1.0. Pellets were washed three times and resuspended with PBS. Ten microliters of suspension was applied on the glow-discharged carbon grids for 60 s, and the excess liquid was blotted with filter paper followed by staining with uranyl acetate for 60 s. The grids were blotted again, rinsed with ultrapure deionized water for 60 s, and then blotted. Once dried, stained grids were examined using a Philips CM100 transmission electron microscope (Philips).

### OMV isolation and characterization.

OMVs derived from four A. pleuropneumoniae strains were isolated by hydrostatic filtration, a dialysis concentration protocol originally described by Musante et al. (56) and adapted by Antenucci et al. (22). Briefly, 50 mL of BHI-NAD broth for each strain was inoculated with overnight cultures to make an initial culture at an OD_600_ value of 0.02 and incubated at 37°C and 200 rpm to late exponential phase (approximately 7 h). Supernatants collected by centrifugation (6,000 × *g*, 20 min, 4°C) were filtered through 0.45-μm sterile filters, then loaded into dialysis membrane columns (Biotech CE dialysis tubing, 1,000-kDa molecular weight cutoff [MWCO], 31-mm flat width; Spectrum Labs), and then sealed with dialysis clips (SnakeSkin dialysis tubing clips; Thermo Scientific). Columns were completely immersed in PBS overnight at 4°C. The next day, a second round of dialysis using fresh PBS (4 h, 4°C) was performed. Finally, the dialyzed samples were concentrated to 200 μL using Amicon Ultra-15 centrifugal filter units (Merck Millipore; MWCO, 10 kDa).

The concentration and size distribution of isolated OMVs were determined by nanoparticle tracking analysis (NTA). OMV samples were diluted in 0.2-μm-filtered PBS and analyzed using a Nanosight NS300 (Malvern Panalytical) and NTA 3.4.003 software (Malvern Panalytical) with the following settings for all samples: camera level of 13, detection threshold of 5, constant flow rate of 10 μL/min, 20 to 140 particles per frame, and five 60-s video captures per sample.

OMV samples for cryo-transmission electron microscopy (cryo-TEM) imaging were prepared as reported previously with minor modifications (57). Briefly, 3 μL extracted OMV suspension was applied on a hydrophilized lacey carbon 300-mesh copper grid (Ted Pella Inc.). The grid was blotted at a blotting time of 5 s, blotting force of 0, temperature of 4°C, and 100% humidity to remove the excess sample and then was rapidly plunged into liquid nitrogen-cooled ethane for sample vitrification (Vitrobot Mark IV; FEI). Sample observations were performed using a Tecnai G2 20 twin transmission electron microscope (FEI) at a voltage of 200 kV under a low-dose rate, and images were recorded with an FEI Eagle camera (4 k by 4 k) at variable nominal magnifications.

### Liquid chromatography-mass spectrometry.

The liquid chromatography-mass spectrometry was performed as previously described (58). Biological duplicates of OMV extracts harvested from each A. pleuropneumoniae strain were diluted to 40 μg of total protein in PBS followed by digestion using 10 μg trypsin (Promega; sequencing grade) overnight at 37°C. Afterward, 5 μL 50% formic acid was added to stop the digestion, and the generated peptides were purified using a ZipTip C_18_ (Merck Millipore) per the manufacturer’s instructions and subsequently dried using a Speed Vac Concentrator Plus (Eppendorf). The tryptic peptides were analyzed using an Ultimate 3000 nano-ultrahigh-performance liquid chromatography (nano-UHPLC) system connected to a Q Exactive mass spectrometer (ThermoFisher Scientific) equipped with a nano-electrospray ion source.

Label-free quantitative mass spectrometry was applied to analyze the protein contents and abundance of OMVs from four strains. All tandem mass spectrometry (MS/MS) data were analyzed using Mascot (Matrix Science, London, UK; version 2.7.0.1). Mascot was set up to search the A. pleuropneumoniae serovar 8 database (4,314 protein entries) following trypsin enzyme digestion. Fragment ion mass tolerance and parent ion tolerance were set to 20 ppm and 10.0 ppm, respectively. Carbamidomethyl of cysteine was specified in Mascot as a fixed modification. Oxidation of methionine and acetyl of the N terminus were specified in Mascot as variable modifications. Scaffold (version Scaffold 5.0.1; Proteome Software Inc., Portland, OR) was used to validate MS/MS-based peptide and protein identifications and determine the abundance of proteins by calculating the normalized spectral abundance factor (NSAF) of each protein (59). Peptide identifications were accepted if they could be established at greater than 95.0% probability by the Scaffold Local FDR algorithm. Protein identifications were accepted if they could be established at greater than 99.0% probability and contained at least two identified peptides. Protein probabilities were assigned by the Protein Prophet algorithm (60). Proteins that contained similar peptides and could not be differentiated based on MS/MS analysis alone were grouped to satisfy the principles of parsimony (61). The subcellular localization and gene ontology (GO) of identified OMV-associated proteins were predicted using PSORTb version 3.0.3 and STRING version 11.5, respectively (62).

### Confocal laser scanning microscopy.

PBS-suspended OMVs were incubated with 5 μL of Vybrant DiI cell-labeling solution (ThermoFisher Scientific) for 30 min at 37°C. To remove excess dye, OMVs were washed twice with 10 mL PBS and reconcentrated with Amicon Ultra-15 centrifugal filter units (10 kDa; Merck Millipore). Then, the protein concentration of labeled OMVs was quantified using a Pierce 660-nm protein assay kit (ThermoFisher Scientific) and adjusted to 100 μg/mL.

The PAM cell line 3D4/31 was purchased from American Type Culture Collection (ATCC, USA). PAMs at 2.5 × 10^5^ were seeded in a four-well imaging chamber (Eppendorf) and allowed to grow overnight. One hundred microliters of DiI-labeled OMVs was added into cell culture and incubated for 4 h. After incubation, cells were washed twice with PBS and incubated with fluorescein-wheat germ agglutinin at a concentration of 10 μg/mL for 15 min at 37°C. Subsequently, cells were washed twice with PBS and then fixed using 3.7% paraformaldehyde for 20 min at room temperature. Then, a few drops of mounting medium supplemented with 4′,6-diamidino-2-phenylindole (DAPI) were added, followed by placement on a glass slide.

Images were captured using a confocal laser scanning microscope (LSM 980 equipped with an Airyscan 2 detector; Carl Zeiss) and processed with ZEN blue software (version 3.4; Carl Zeiss). Fluorescein was visualized with a 488-nm laser, DAPI with a 405-nm laser, and DiI with a 561-nm laser.

### Cytotoxicity assessment of A. pleuropneumoniae OMVs on PAMs.

The cytotoxicity of OMVs to PAMs was determined by measuring the released cytosolic lactate dehydrogenase (LDH) in the cell supernatant. PAMs were seeded into 24-well plates at a density of 2 × 10^6^ cells per well followed by overnight growth to establish 90% confluence. Afterward, the spent medium was discarded and replaced with fresh phenol red-free RPMI 1640 medium without fetal bovine serum (FBS). In the pilot experiments, cells were incubated with 5 μg/mL, 10 μg/mL, and 20 μg/mL OMVs per well for 6 h, 12 h, and 24 h, respectively. According to the results, 20 μg/mL OMVs and 24 h of incubation time were used for subsequent experiments. Cells incubated with A. pleuropneumoniae live bacteria at a multiplicity of infection (MOI) of 1:10 were set up as positive controls. After incubation, the cell supernatants were clarified by centrifugation, and 50 μL of supernatant was transferred to a 96-well plate for LDH assay using the CytoTox 96 nonradioactive cytotoxicity assay kit (Promega) per the manufacturer’s instructions. Cells treated with PBS were used as 100% live cell control, and cells lysed with 1% (wt/vol) Triton X were used as maximum LDH release control. The percentage of cytotoxicity was calculated as (stimulated cell release − PBS-treated cell release)/(maximum release − PBS-treated cell release) × 100.

For the viability assay, PAMs were seeded into 96-well plates at a density of 1.5 × 10^5^ cells per well and let grow overnight until 90% confluence. Then, the spent culture medium was replaced with 100 μL of phenol red-free RPMI 1640 containing 20 μg/mL OMVs or A. pleuropneumoniae live cells at an MOI of 1:10 for 24 h. Afterward, cells were washed twice with PBS and incubated with 100 μL of fresh medium containing 10% PrestoBlue HS cell viability reagent (ThermoFisher Scientific) for 30 min at 37°C. PBS-treated cells were regarded as 100% viability, and cells lysed with 1% (wt/vol) Triton X after incubation were regarded as 0% viability. Fluorescence was measured using an excitation wavelength of 560 nm and an emission wavelength of 590 nm (Synergy H1 microplate reader; BioTek). Percentage of viability was calculated as (fluorescence of OMV- or bacterium-treated cells − fluorescence of 1% Triton X-treated cells)/(fluorescence of PBS-treated cells − fluorescence of 1% Triton X-treated cells) × 100.

### Gene expression determination by high-throughput quantitative real-time PCR.

**(i) Cell stimulation and RNA extraction.** PAMs at 2 × 10^6^ were seeded into 24-well plates overnight and stimulated with 20 μg/mL OMVs or inactivated A. pleuropneumoniae cells (100 μg/mL gentamicin for 1 h) at a 1:10 MOI for 24 h. As well, PBS and commercial lipopolysaccharide (LPS) from Escherichia coli O111:B4 (Sigma-Aldrich) were used to stimulate PAMs as negative and positive controls, respectively. Subsequently, total RNA was extracted from stimulated PAMs using the RNeasy minikit (Qiagen). The quantity and purity of extracted RNA were determined by a NanoDrop 1000 spectrophotometer (ThermoFisher Scientific). The samples had an average concentration of 190.9 ± 24.3 ng/μL and an average *A*_260_/*A*_280_ ratio of 2.1 ± 0.01. The quality of RNA was assessed using an Agilent Bioanalyzer to assign an RNA integrity number (RIN) to each sample. The samples had a high average RIN value of 9.0 ± 0.8, which was considered adequate for downstream analyses.

**(ii) cDNA synthesis, preamplification, and exonuclease treatment.** Duplicate cDNA synthesis was made from 500 ng total RNA of each sample using the QuantiTect reverse transcription kit per the manufacturer’s instructions (Qiagen). Two RNA samples were randomly selected to prepare additional non-reverse transcriptase controls. Synthesized cDNA was diluted 10-fold in low-EDTA Tris-EDTA (TE) buffer (PanReac AppliChem), and 2.5 μL of diluted cDNA was used for preamplification using TaqMan PreAmp master mix (Applied Biosystems). PreAmp master mix at 3 μL, low-EDTA TE buffer at 2 μL, cDNA at 2.5 μL, and 2.5 μL of a 200 nM primer mixture were mixed and incubated under the conditions of 95°C for 10 min followed by 14 cycles of 95°C for 15 s and 60°C for 4 min. All preamplified cDNA was treated with 16 U exonuclease I (New England BioLabs) for 30 min and 10-fold diluted in low-EDTA TE buffer for qPCR analysis.

**(iii) Microfluidic high-throughput qPCR.** qPCR was carried out on a 96.96 dynamic array integrated fluid circuit chip (Fluidigm) in the Biomark HD system under the following conditions: 50°C for 2 min and 95°C for 10 min followed by 35 cycles of 95°C for 15 s and 60°C for 1 min. Melting curves were generated after PCR running (from 60°C to 95°C, increasing 1°C/3 s). Nontemplate controls and non-reverse transcriptase controls were included on the chip to inspect contamination, nonspecific amplification, and genomic DNA interference. Three dilution series of preamplified cDNA pools were used to construct standard curves for each pair of primers to determine the efficiency of amplification and dynamic range of the different qPCRs. A total of 63 genes, including 5 reference genes, were included in the experiment. Subsequently, 22 genes were excluded from the data analysis due to quality control of melting curves, standard curves, and non-reverse transcriptase controls. All primers used for microfluidic qPCR are listed in Table S4.

**(iv) Microfluidic qPCR data analysis.** Raw data were preprocessed using the Fluidigm real-time PCR analysis software 4.7.1 (Fluidigm). Melting curves were inspected to confirm the presence of a single PCR product, and amplification efficiencies were calculated based on the standard curves for each primer pair. Quantification cycle (*C_q_*) values acquired using Fluidigm real-time PCR analysis software were exported to GenEx5 software (MultiD) for data preprocessing, including correction for PCR efficiency, normalization to reference genes, averaging of cDNA technical repeats, and calculation of relative quantification values. Out of 5 reference genes, actin beta (ACTB), beta-2-microglobulin (B2M), and glyceraldehyde-3-phosphate (GAPDH) were found to be the most stably expressed and thus were used for data normalization. Kyoto Encyclopedia of Genes and Genomes (KEGG) pathway enrichment analysis was performed using STRING version 11.5 (63).

### qPCR for detecting expression of genes in PAMs.

To investigate the possible inhibitory effect of WT OMVs on the immune responses of PAMs, pretreatment and cotreatment of PAMs with OMVs and WT cells were characterized. For pretreatment, 2 × 10^6^ PAMs were seeded into 24-well plates overnight and then treated with 0.2 μg/mL, 2 μg/mL, and 20 μg/mL OMVs for 24 h, and then cells were washed with PBS and stimulated with inactivated WT cells (100 μg/mL gentamicin for 1 h) at an MOI of 1:10 for another 24 h. For cotreatment, PAMs were treated with OMVs, followed by infection with either inactivated WT cells or live WT cells at an MOI of 1:10 immediately. Subsequently, cells were subjected to RNA extraction, reverse transcription, and qPCR as described under “Characterization of A. pleuropneumoniae strains.” The relative expression level was normalized to the *GAPDH* gene, and the results were calculated by the 2^−ΔΔ^*^CT^* method. Primer sequences can be found in Table S4.

### Statistical analysis.

All statistical analyses were performed using the GraphPad Prism 8 software (GraphPad Software Inc.). Statistical significance was analyzed using one-way analysis of variance (ANOVA) (Dunnett’s correction for multiple comparison). For the autoaggregation assay, area under the curve was calculated and subjected to one-way ANOVA, followed by Dunnett’s correction for multiple comparison to determine the statistical significance between individual strains.
