# Supplementary material for: Outer Membrane Vesicles of Actinobacillus pleuropneumoniae Exert Immunomodulatory Effects on Porcine Alveolar Macrophages
Source: Microbiol Spectr. 2022 Aug 30;10(5):e01819-22. doi: 10.1128/spectrum.01819-22 (PMC9602539; doi:10.1128/spectrum.01819-22)
Supplement: Supplemental file 1 — Fig. S1 to S7 and Tables S1 to S4. Download spectrum.01819-22-s0001.pdf, PDF file, 1.6 MB [file spectrum.01819-22-s0001.pdf]

Supplementary figures and tables

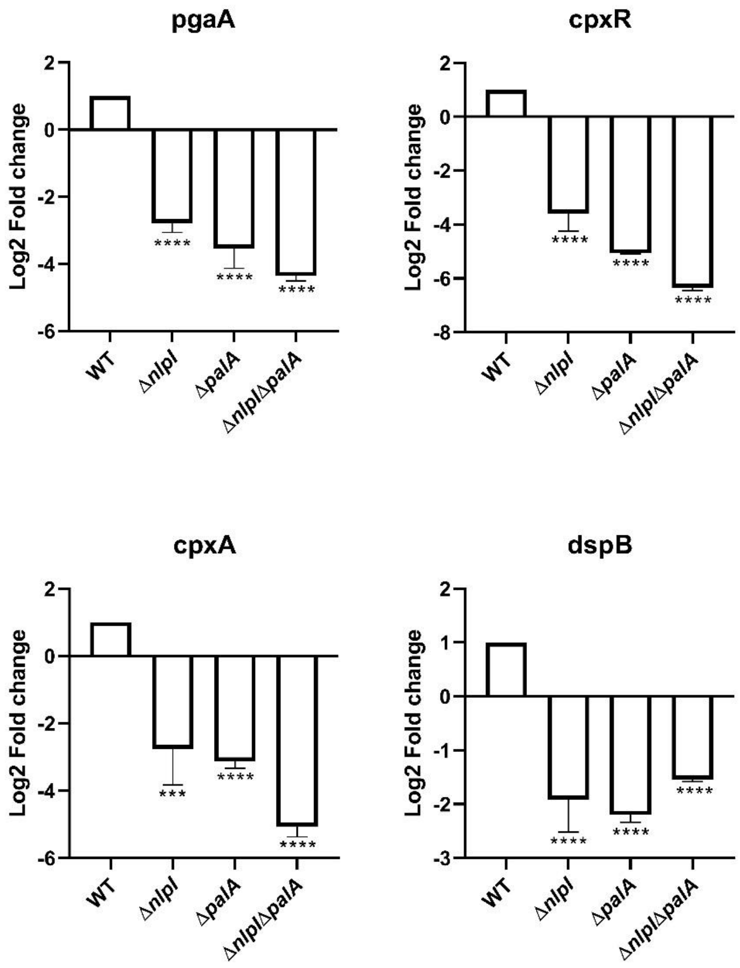

Fig. S1. The expression levels of genes involved in biofilm formation in *A. pleuropneumoniae* were determined by qPCR. Data represent means ± SD of log2 fold change values calculated from biological triplicates. Statistically significant differences compared to the WT group are indicated with asterisks (***$p < 0.001$, ****$p < 0.0001$).

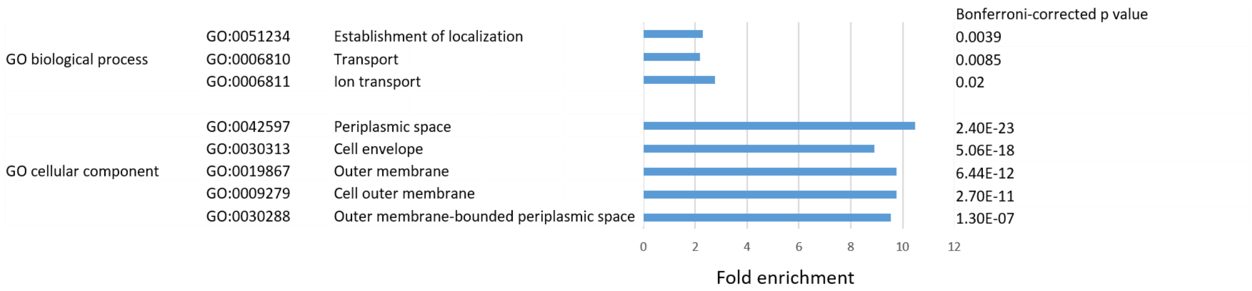

Fig. S2. Gene ontology (GO) enrichment analysis of WT OMV-associated proteins was performed using STRING. Only categories with a Bonferroni-corrected *p* value < 0.05 were shown in the graph.

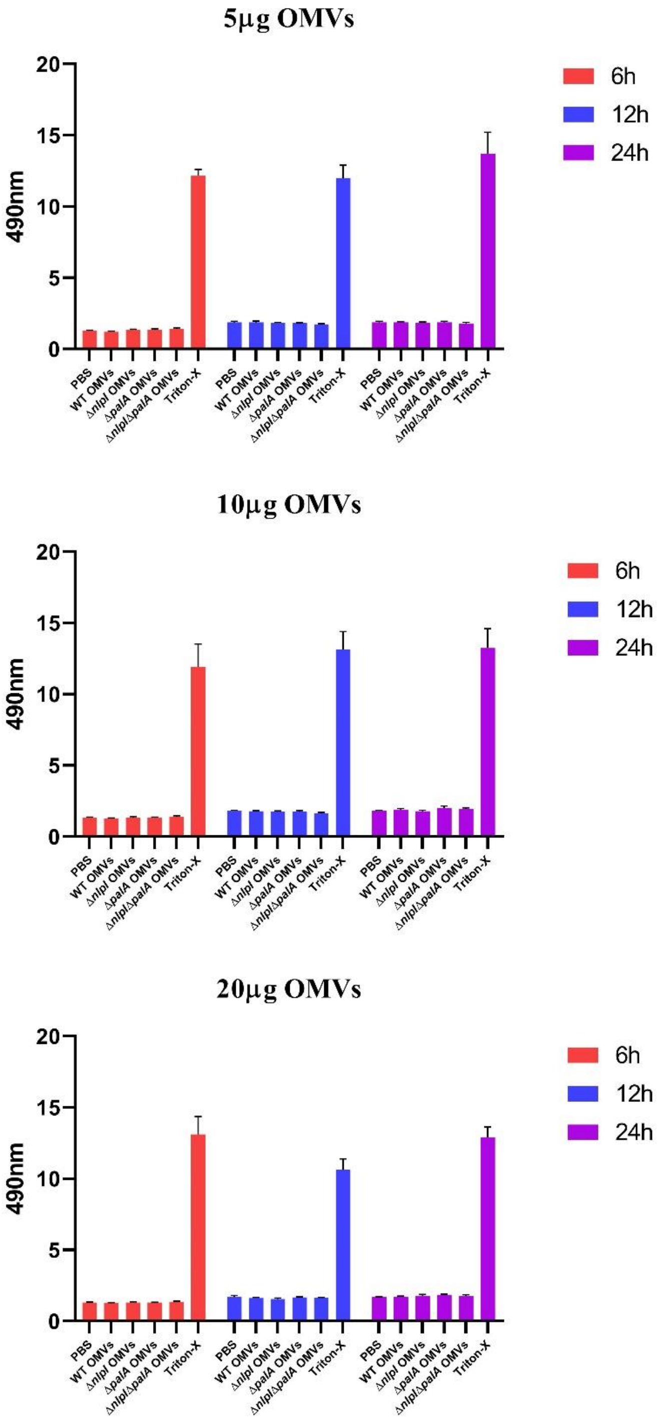

Fig. S3. Optimization of OMV concentrations and incubation time for cytotoxicity assay. PAMs were incubated with different concentrations of OMVs for different time periods. Cytotoxicity was determined by measuring LDH released into the culture supernatant after incubation. PBS-treated group and 1% (w/v) Triton-X-treated group were used as negative and positive controls, respectively.

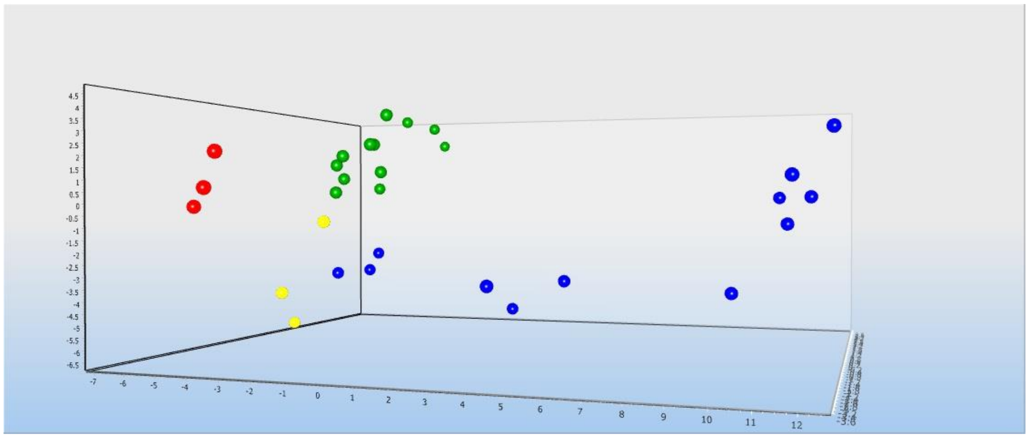

Fig. S4. Principle component analysis of the relative expression of all genes analyzed from different treatment groups: PBS-treated groups (red dots), four OMV-treated groups (green dots), four bacterial cell-treated groups (blue dots), and *E. coli* LPS-treated groups (yellow).

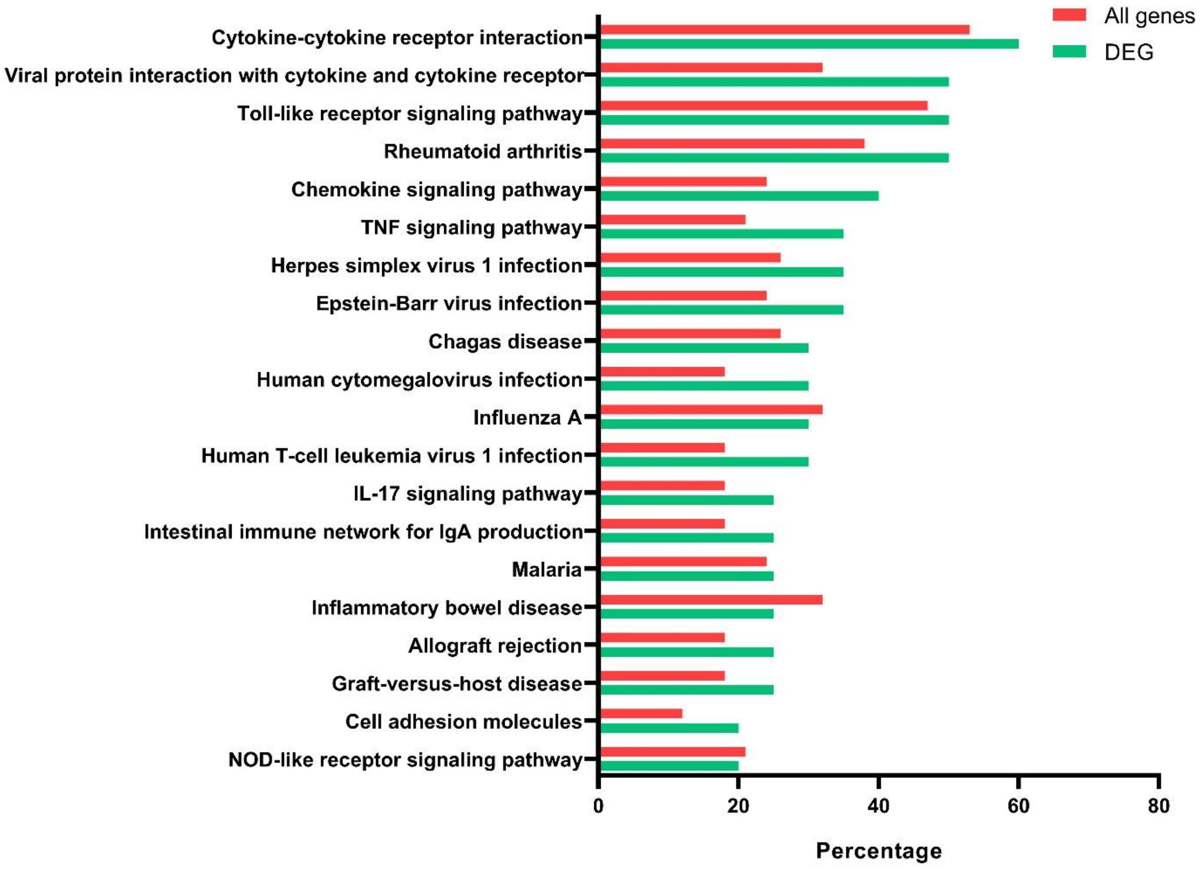

Fig. S5. KEGG pathway enrichment analysis of 20 differentially expressed genes (DEGs) found in inactivated WT and Δ*nlpI* bacterial cells-treated PAMs. A total of 35 genes included in high-throughput RT-PCT analysis were used as background for KEGG enrichment analysis, except for housekeeping genes and IL23 which cannot be mapped in the database. Chi-square test was used for statistical significance analysis of enrichment between background genes and DEGs in each pathway.

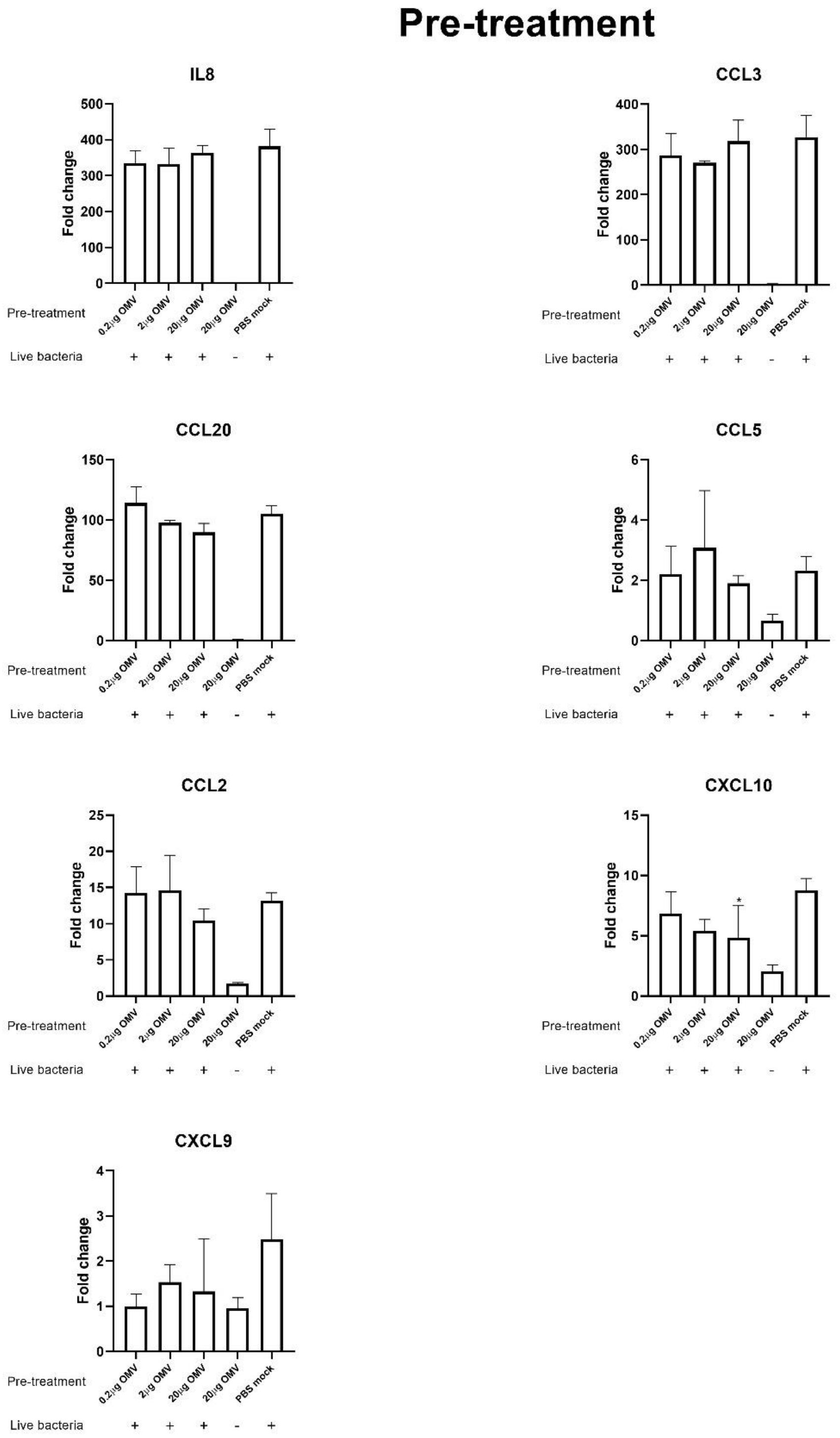

Fig. S6. Pre-treatment of PAMs with different doses of OMVs for 24 h prior to stimulation of live WT bacterial cells for another 24 h was not able to significantly reduce the expression of cytokines compared to PAMs stimulated with live WT bacterial cells alone, except for CXCL10.

Valid	...	Sequence	Prob	Masc...	Masc...	Masc...	NTT	Intensity	Modifications	TIC	Observed	Start	Actual Mass	Stop	Charge	# Ot...	Delta ...	Other Prot...	Delta ...	Spectrum ID	Rete...
☑	✓	(M)STWSSMLADLK(K)	100%	47.2	25.0	47.2	1		Oxidation (+16)	411700	627.80	2	1,253.59	12	2	0	-0.0018		-1.5	CJK_210215_E2;...	3610
☑	✓	(M)STWSSMLADLK(K)	100%	73.9	25.0	73.9	1			1446000	619.81	2	1,237.60	12	2	0	-0.00085		-0.69	CJK_210215_E2;...	5050
☑	✓	(M)STWSSMLADLK(K)	100%	26.5	25.0	26.5	1			303800	619.81	2	1,237.60	12	2	0	-0.0023		-1.9	CJK_210215_E2;...	5060
☑	✓	(M)STWSSMLADLKK(R)	100%	48.3	25.0	48.3	1			2306000	456.24	2	1,365.70	13	3	0	0.00020		0.15	CJK_210215_E2;...	4040
☑	✓	(M)STWSSMLADLKK(R)	100%	41.7	25.0	41.7	1			2438000	456.24	2	1,365.70	13	3	0	-0.000...		-0.0095	CJK_210215_E2;...	4060
☑	✓	(M)STWSSMLADLKK(R)	100%	38.4	25.0	38.4	1		Oxidation (+16)	712000	461.57	2	1,381.69	13	3	0	-0.0013		-0.97	CJK_210215_E2;...	2670
☑	✓	(M)STWSSMLADLKK(R)	100%	35.9	25.0	35.9	1			826700	683.86	2	1,365.70	13	2	0	0.0013		0.97	CJK_210215_E2;...	4050
☑	✓	(M)STWSSMLADLKK(R)	100%	26.9	25.0	26.9	1			323000	456.24	2	1,365.70	13	3	0	0.00078		0.57	CJK_210215_E2;...	4080
☑	✓	(M)STWSSMLADLKK(R)	100%	19.1	25.0	19.1	1		Oxidation (+16)	947200	461.57	2	1,381.69	13	3	0	-0.00058		-0.42	CJK_210215_E2;...	2680
☑	✓	(K)NGLQVGVSQAK(L)	100%	39.1	25.0	39.1	2			147600	582.80	31	1,163.59	41	2	0	0.00069		0.59	CJK_210215_E2;...	1490
☑	✓	(K)NGLQVGVSQAK(L)	100%	27.3	25.0	27.3	2			89280	582.80	31	1,163.59	41	2	0	-0.00094		-0.81	CJK_210215_E2;...	1480
☑	✓	(K)YGNKLVLVIPK(E)	99%	13.8	25.0	13.8	2			581800	415.60	54	1,243.77	64	3	0	1.0		1.5	CJK_210215_E2;...	3090
☑	✓	(K)EYDGSVGNGFFDLVK(A)	100%	27.9	25.0	27.9	2			165700	823.89	65	1,645.76	79	2	0	-0.0012		-0.73	CJK_210215_E2;...	5360
☑	✓	(K)AAEELGIQVK(Y)	100%	58.3	25.0	58.3	2			514600	529.30	80	1,056.58	89	2	0	0.000066		0.062	CJK_210215_E2;...	2220
☑	✓	(K)AAEELGIQVK(Y)	100%	34.2	25.0	34.2	2			270200	529.30	80	1,056.58	89	2	0	-0.0013		-1.2	CJK_210215_E2;...	2260
☑	✓	(K)AAEELGIQVK(Y)	100%	70.1	25.0	70.1	2			1418000	529.30	80	1,056.58	89	2	0	0.00022		0.20	CJK_210215_E2;...	2240
☑	✓	(K)YVNRNELEYAHK(S)	100%	29.4	25.0	29.4	2			404700	491.26	90	1,470.75	101	3	0	-0.0053		-3.6	CJK_210215_E2;...	940
☑	✓	(K)SLGTADQFLGLTER(G)	100%	52.8	25.0	52.8	2			382100	754.39	102	1,506.76	115	2	0	-0.0059		-3.9	CJK_210215_E2;...	4470
☑	✓	(K)SLGTADQFLGLTER(G)	100%	54.5	25.0	54.5	2			357700	754.39	102	1,506.77	115	2	0	-0.0011		-0.75	CJK_210215_E2;...	4520
☑	✓	(K)SLGTADQFLGLTER(G)	100%	93.4	25.0	93.4	2			420100	754.39	102	1,506.77	115	2	0	-0.00098		-0.65	CJK_210215_E2;...	4500
☑	✓	(K)SLGTADQFLGLTER(G)	100%	99.8	25.0	99.8	2			3461000	754.39	102	1,506.77	115	2	0	-0.0016		-1.1	CJK_210215_E2;...	4490
☑	✓	(R)GLTLFAPQLDQFLQK(H)	100%	51.7	25.0	51.7	2			2740000	573.65	116	1,717.94	130	3	0	0.0018		1.0	CJK_210215_E2;...	6450
☑	✓	(R)GLTLFAPQLDQFLQK(H)	100%	56.9	25.0	56.9	2			1.279E7	859.98	116	1,717.94	130	2	0	-0.0016		-0.91	CJK_210215_E2;...	6450
☑	✓	(R)GLTLFAPQLDQFLQK(H)	100%	46.4	25.0	46.4	2			430400	859.98	116	1,717.94	130	2	0	-0.00064		-0.37	CJK_210215_E2;...	6480
☑	✓	(R)GLTLFAPQLDQFLQK(H)	100%	45.3	25.0	45.3	2			577300	573.65	116	1,717.94	130	3	0	0.0015		0.87	CJK_210215_E2;...	6440
☑	✓	(R)GLTLFAPQLDQFLQK(H)	100%	40.1	25.0	40.1	2			2123000	859.98	116	1,717.94	130	2	0	-0.0012		-0.72	CJK_210215_E2;...	6460
☑	✓	(R)GLTLFAPQLDQFLQK(H)	100%	39.1	25.0	39.1	2			351400	573.65	116	1,717.94	130	3	0	0.0020		1.2	CJK_210215_E2;...	6470
☑	✓	(K)ISNVVGSSTGDAVSK(L)	100%	55.4	25.0	55.4	2			306200	474.25	134	1,419.72	148	3	0	0.0020		1.4	CJK_210215_E2;...	1460
☑	✓	(K)ISNVVGSSTGDAVSK(L)	100%	45.1	25.0	45.1	2			323200	710.87	134	1,419.72	148	2	0	-0.0012		-0.82	CJK_210215_E2;...	1450
☑	✓	(K)ISNVVGSSTGDAVSK(L)	100%	71.3	25.0	71.3	2			1400000	710.87	134	1,419.72	148	2	0	-0.0012		-0.85	CJK_210215_E2;...	1470
☑	✓	(K)ISNVVGSSTGDAVSK(L)	100%	18.4	25.0	18.4	2			113200	710.87	134	1,419.72	148	2	0	-0.00069		-0.49	CJK_210215_E2;...	1480
☑	✓	(K)GTTTIDAFTTQIQNFGK(L)	100%	56.3	25.0	56.3	2			1729000	614.98	203	1,841.92	219	3	0	-0.00036		-0.20	CJK_210215_E2;...	5110
☑	✓	(K)GTTTIDAFTTQIQNFGK(L)	100%	58.5	25.0	58.5	2			128700	921.97	203	1,841.92	219	2	0	0.00027		0.15	CJK_210215_E2;...	5120
☑	✓	(K)GTTTIDAFTTQIQNFGK(L)	100%	42.7	25.0	42.7	2			130600	921.96	203	1,841.91	219	2	0	-0.0020		-1.1	CJK_210215_E2;...	5090
☑	✓	(K)GTTTIDAFTTQIQNFGK(L)	100%	88.7	25.0	88.7	2			1417000	921.96	203	1,841.91	219	2	0	-0.0015		-0.79	CJK_210215_E2;...	5110
☑	✓	(K)GTTTIDAFTTQIQNFGK(L)	100%	25.8	25.0	25.8	2			217600	614.98	203	1,841.92	219	3	0	0.000042		0.023	CJK_210215_E2;...	5120
☑	✓	(R)QLQNISGSALSK(T)	100%	47.5	25.0	47.5	2			355400	623.34	233	1,244.67	244	2	0	0.000012		0.0097	CJK_210215_E2;...	1840
☑	✓	(R)QLQNISGSALSK(T)	100%	42.1	25.0	42.1	2			2360000	623.34	233	1,244.67	244	2	0	-0.00048		-0.38	CJK_210215_E2;...	1820
☑	✓	(R)QLQNISGSALSK(T)	96%	9.1	25.0	9.1	2			216500	623.34	233	1,244.67	244	2	0	-0.0024		-1.9	CJK_210215_E2;...	1810
☑	✓	(K)TGLGLDIISSLLSGVTASFALANK(N)	100%	90.0	25.0	90.0	2			7113000	783.44	245	2,347.30	268	3	0	-0.0049		-2.1	CJK_210215_E2;...	6880
☑	✓	(K)TGLGLDIISSLLSGVTASFALANK(N)	100%	112.5	25.0	112.5	2			1.213E7	1,174.66	245	2,347.30	268	2	0	0.0034		1.4	CJK_210215_E2;...	6880
☑	✓	(K)TGLGLDIISSLLSGVTASFALANKNASTSTK	100%	114.8	25.0	114.8	2			2853000	1,013.55	245	3,037.64	275	3	0	1.0		0.72	CJK_210215_E2;...	6870
☑	✓	(K)YAAGFELSNQVIGGITK(A)	100%	52.7	25.0	52.7	2			290000	852.47	276	1,702.93	292	2	0	0.00020		0.12	CJK_210215_E2;...	4690
☑	✓	(K)YAAGFELSNQVIGGITK(A)	100%	57.0	25.0	57.0	2			555500	568.65	276	1,702.93	292	3	0	-0.00025		-0.15	CJK_210215_E2;...	4670
☑	✓	(K)YAAGFELSNQVIGGITK(A)	100%	86.9	25.0	86.9	2			2084000	852.47	276	1,702.92	292	2	0	-0.0011		-0.63	CJK_210215_E2;...	4670
☑	✓	(K)YAAGFELSNQVIGGITK(A)	99%	12.3	25.0	12.3	2			78970	852.47	276	1,702.93	292	2	0	0.0015		0.88	CJK_210215_E2;...	4700
☑	✓	(K)YAAGFELSNQVIGGITK(A)	98%	12.7	25.0	12.7	2			93890	568.98	276	1,703.93	292	3	0	1.00		-1.7	CJK_210215_E2;...	4690
☑	✓	(K)AVSSYILAQR(L)	100%	59.2	25.0	59.2	2			1157000	554.31	293	1,106.61	302	2	0	0.0000...	BAV25209.1...	0.0073	CJK_210215_E2;...	2590
☑	✓	(K)AVSSYILAQR(L)	100%	59.3	25.0	59.3	2			1.992E7	554.31	293	1,106.61	302	2	0	0.00040	BAV25209.1...	0.36	CJK_210215_E2;...	2560
☑	✓	(K)AVSSYILAQR(L)	100%	65.1	25.0	64.3	2			3799000	554.31	293	1,106.61	302	2	0	0.00064	BAV25209.1...	0.58	CJK_210215_E2;...	2570
☑	✓	(K)AVSSYILAQR(L)	100%	41.2	25.0	37.2	2			114700	554.31	293	1,106.61	302	2	0	-0.0011	BAV25209.1...	-1.0	CJK_210215_E2;...	2630
☑	✓	(K)AVSSYILAQR(L)	100%	70.0	25.0	66.9	2			500300	554.31	293	1,106.61	302	2	0	-0.000...	BAV25209.1...	-0.040	CJK_210215_E2;...	2600
☑	✓	(K)AVSSYILAQR(L)	100%	34.8	25.0	32.0	2			241400	554.31	293	1,106.61	302	2	0	-0.00040	BAV25209.1...	-0.36	CJK_210215_E2;...	2620
☑	✓	(K)AVSSYILAQR(L)	98%	8.9	25.0	6.9	2			70160	554.31	293	1,106.61	302	2	0	-0.00061	BAV25209.1...	-0.55	CJK_210215_E2;...	2670
☑	✓	(R)LAAGLSTTGPAAALIASSISLAISPLAFLR(\	100%	84.7	25.0	84.7	2			5.258E7	951.89	303	2,852.64	332	3	0	-0.0010		-0.36	CJK_210215_E2;...	6880
☑	✓	(R)SKEIGEFAER(F)	100%	14.7	25.0	14.7	2			80350	583.30	340	1,164.58	349	2	0	-0.00020		-0.17	CJK_210215_E2;...	1210
☑	✓	(K)EIGEFAER(F)	99%	36.8	25.0	36.8	2			146100	475.73	342	949.45	349	2	0	0.000088		0.093	CJK_210215_E2;...	1820
☑	✓	(K)EIGEFAER(F)	99%	35.0	25.0	35.0	2			297600	475.73	342	949.45	349	2	0	-0.000...		-0.036	CJK_210215_E2;...	1800
☑	✓	(K)NYFENGYDAR(H)	100%	21.5	25.0	21.5	2			99400	624.77	446	1,247.52	455	2	0	0.0019		1.5	CJK_210215_E2;...	2210
☑	✓	(R)HKAFLEDSFSLLSSFNK(Q)	100%	58.9	25.0	58.9	2			605000	657.34	456	1,968.99	472	3	0	-0.000...		-0.049	CJK_210215_E2;...	4960
☑	✓	(R)HKAFLEDSFSLLSSFNK(Q)	100%	37.1	25.0	37.1	2			78630	657.34	456	1,968.99	472	3	0	-0.00071		-0.36	CJK_210215_E2;...	5010
☑	✓	(R)HKAFLEDSFSLLSSFNK(Q)	100%	69.2	25.0	69.2	2			290500	657.34	456	1,968.99	472	3	0	0.000095		0.048	CJK_210215_E2;...	4970
☑	✓	(R)HKAFLEDSFSLLSSFNK(Q)	100%	34.8	25.0	32.8	2			177700	493.26	456	1,969.00	472	4	0	0.0011		0.56	CJK_210215_E2;...	4960
☑	✓	(R)HKAFLEDSFSLLSSFNK(Q)	100%	18.8	25.0	18.8	2			101400	493.26	456	1,969.00	472	4	0	0.0023		1.2	CJK_210215_E2;...	4970
☑	✓	(K)AFLEDSFSLLSSFNK(Q)	100%	43.4	25.0	43.4	2			168000	852.93	458	1,703.84	472	2	0	0.00016		0.092	CJK_210215_E2;...	6630
☑	✓	(K)AFLEDSFSLLSSFNK(Q)	100%	68.0	25.0	68.0	2			1159000	568.95	458	1,703.84	472	3	0	-0.00097		-0.57	CJK_210215_E2;...	6600
☑	✓	(K)AFLEDSFSLLSSFNK(Q)	100%	30.0	25.0	30.0	2			112700	852.93	458	1,703.84	472	2	0	0.00086		0.50	CJK_210215_E2;...	6650
☑	✓	(K)AFLEDSFSLLSSFNK(Q)	100%	75.2	25.0	75.2	2			186500	852.93	458	1,703.84	472	2	0	-0.0026		-1.5	CJK_210215_E2;...	6580
☑	✓	(K)AFLEDSFSLLSSFNK(Q)	100%	108.7	25.0	108.7	2			671500	852.93	458	1,703.84	472	2	0	-0.00098		-0.57	CJK_210215_E2;...	6620
☑	✓	(K)AFLEDSFSLLSSFNK(Q)	100%	122.8	25.0	122.8	2			5284000	852.93	458	1,703.84	472	2	0	-0.0016		-0.92	CJK_210215_E2;...	6600
☑	✓	(K)AFLEDSFSLLSSFNK(Q)	99%	11.2	25.0	11.2	2			101000	568.95	458	1,703.84	472	3	0	0.00054		0.32	CJK_210215_E2;...	6590
☑	✓	(K)AFLEDSFSLLSSFNKQYETER(A)	100%	21.4	25.0	21.4	2			82260	838.07	458	2,511.19	478	3	0	1.00		-2.2	CJK_210215_E2;...	6120
☑	✓	(R)AYLITQQR(W)	99%	45.5	25.0	45.5	2			2660000	464.78	479	927.55	486	2	0	0.00030		0.32	CJK_210215_E2;...	1370
☑	✓	(R)AYLITQQR(W)	99%	42.6	25.0	42.6	2			1068000	464.78	479	927.55	486	2	0	0.00022		0.24	CJK_210215_E2;...	1390
☑	✓	(R)AYLITQQR(W)	99%	23.2	25.0	23.2	2			402400	464.78	479	927.55	486	2	0	0.00080		0.86	CJK_210215_E2;...	1400
☑	✓	(R)AYLITQQR(W)	99%	14.0	25.0	14.0	2			241200	464.78	479	927.55	486	2	0	0.0016		1.7	CJK_210215_E2;...	1350
☑	✓	(R)WDEYIGELAGITGK(G)	100%	66.9	25.0	66.9	2			727400	776.39	487	1,550.76	500	2	0	-0.0012		-0.78	CJK_210215_E2;...	5260
☑	✓	(R)WDEYIGELAGITGK(G)	100%	91.4	25.0	91.4	2			1169000	776.39	487	1,550.76	500	2	0	-0.0017		-1.1	CJK_210215_E2;...	5250
☑	✓	(R)WDEYIGELAGITGKGDK(L)	100%	52.9	25.0	52.9	2			409900	617.98	487	1,850.91	503	3	0	0.0000...		0.000031	CJK_210215_E2;...	4460
☑	✓	(R)WDEYIGELAGITGKGDK(L)	100%	53.7	25.0	53.7	2			268400	617.98	487	1,850.91	503	3	0	0.00048		0.26	CJK_210215_E2;...	4440
☑	✓	(R)WDEYIGELAGITGKGDK(L)	100%	14.3	25.0	14.3	2			50170	617.98	487	1,850.90	503	3	0	-0.00088		-0.48	CJK_210215_E2;...	4470
☑	✓	(K)AYVDYFQEGK(L)	100%	47.7	25.0	47.7	2			2227000	610.28	509	1,218.56	518	2	0	-0.00046		-0.38	CJK_210215_E2;...	2930
☑	✓	(K)AYVDYFQEGK(L)	100%	30.6	25.0	30.6	2			313400	610.29	509	1,218.56	518	2	0	0.0023		1.9	CJK_210215_E2;...	2910
☑	✓	(K)AYVDYFQEGK(L)	100%	27.5	25.0	27.5	2			409800	610.29	509	1,218.56	518	2	0	0.00016		0.13	CJK_210215_E2;...	2940
☑	✓	(K)LLEKKPDDFSK(V)	100%	24.6	25.0	24.6	2			187600	440.58	519	1,318.71	529	3	0	-0.00012		-0.091	CJK_210215_E2;...	1010
☑	✓	(K)VVFDPTKGEIDISNSQTSTLLK(F)	100%	29.0	25.0	29.0	2			85340	798.09	530	2,391.25	551	3	0	-0.0018		-0.77	CJK_210215_E2;...	4590
☑	✓	(K)VVFDPTKGEIDISNSQTSTLLK(F)	100%	81.3	25.0	81.3	2			3194000	798.09	530	2,391.25	551	3	0	-0.0016		-0.68	CJK_210215_E2;...	4560
☑	✓	(K)VVFDPTKGEIDISNSQTSTLLK(F)	100%	90.1	25.0	90.1	2			831700	798.09	530	2,391.25	551	3	0	-0.0011		-0.48	CJK_210215_E2;...	4580
☑	✓	(K)FVTPLLTPGTESR(E)	100%	46.5	25.0	46.5	2			1490000	709.39	552	1,416.76	564	2	0	-0.00098		-0.69	CJK_210215_E2;...	3880
☑	✓	(K)FVTPLLTPGTESR(E)	100%	45.8	25.0	45.8	2			220000	473.26	552	1,416.76	564	3	0	-0.0021		-1.5	CJK_210215_E2;...	3870
☑	✓	(K)FVTPLLTPGTESR(E)	100%	45.3	25.0	45.3	2			4228000	709.39	552	1,416.76	564	2	0	-0.0010		-0.71	CJK_210215_E2;...	3850
☑	✓	(K)FVTPLLTPGTESR(E)	100%	62.7	25.0	62.5	2			7083000	709.39	552	1,416.76	564	2	0	-0.00096		-0.67	CJK_210215_E2;...	3870
☑	✓	(K)FVTPLLTPGTESR(E)	100%	37.3	25.0	36.4	2			406600	709.89	552	1,417.76	564	2	0	1.00		-3.0	CJK_210215_E2;...	3900
☑	✓	(K)FVTPLLTPGTESR(E)	100%	28.5	25.0	28.5	2			546000	709.39	552	1,416.76	564	2	0	-0.0011		-0.79	CJK_210215_E2;...	3900
☑	✓	(K)FVTPLLTPGTESR(E)	96%	7.5	25.0	7.5	2			105100	473.59	552	1,417.76	564	3	0	1.0		-1.3	CJK_210215_E2;...	3890
☑	✓	(K)DKGAVYDYTNLIQHAHISSSVAR(G)	100%	42.3	25.0	42.3	2			401600	637.08	593	2,544.28	615	4	0	0.0066		2.6	CJK_210215_E2;...	3720
☑	✓	(K)DKGAVYDYTNLIQHAHISSSVAR(G)	100%	81.8	25.0	81.8	2			2480000	637.08	593	2,544.27	615	4	0	-0.000...		-0.037	CJK_210215_E2;...	3700
☑	✓	(K)DKGAVYDYTNLIQHAHISSSVAR(G)	100%	25.5	25.0	25.5	2			293100	510.06	593	2,545.27	615	5	0	1.0		-0.66	CJK_210215_E2;...	3700
☑	✓	(K)DKGAVYDYTNLIQHAHISSSVAR(G)	100%	24.7	25.0	24.7	2			87700	849.10	593	2,544.27	615	3	0	0.0022		0.87	CJK_210215_E2;...	3710
☑	✓	(K)DKGAVYDYTNLIQHAHISSSVAR(G)	95%	7.2	25.0	7.2	2			153900	637.07	593	2,544.27	615	4	0	-0.0039		-1.5	CJK_210215_E2;...	3690
☑	✓	(K)GAVYDYTNLIQHAHISSSVAR(G)	100%	56.0	25.0	56.0	2			84050	768.06	595	2,301.15	615	3	0	-0.0033		-1.4	CJK_210215_E2;...	3850
☑	✓	(K)GAVYDYTNLIQHAHISSSVAR(G)	100%	59.5	25.0	59.5	2			1974000	576.30	595	2,301.15	615	4	0	0.00077		0.33	CJK_210215_E2;...	3830
☑	✓	(K)GAVYDYTNLIQHAHISSSVAR(G)	100%	38.5	25.0	38.5	2			232000	576.30	595	2,301.15	615	4	0	0.00090		0.39	CJK_210215_E2;...	3850
☑	✓	(K)GAVYDYTNLIQHAHISSSVAR(G)	100%	71.1	25.0	71.1	2			660000	768.06	595	2,301.15	615	3	0	-0.00092		-0.40	CJK_210215_E2;...	3830
☑	✓	(K)GAVYDYTNLIQHAHISSSVAR(G)	100%	31.3	25.0	31.3	2			677800	576.29	595	2,301.15	615	4	0	-0.00092		-0.40	CJK_210215_E2;...	3820
☑	✓	(R)DYELTQSGNSNLK(A)	100%	19.3	25.0	19.3	2			150000	734.85	711	1,467.68	723	2	0	-0.0010		-0.68	CJK_210215_E2;...	2050
☑	✓	(K)AHDELHSVEEIIGSNQR(D)	100%	45.5	25.0	45.5	2			260800	484.24	724	1,932.93	740	4	0	0.0012		0.62	CJK_210215_E2;...	3120
☑	✓	(K)AHDELHSVEEIIGSNQR(D)	100%	63.0	25.0	63.0	2			379900	645.32	724	1,932.93	740	3	0	-0.0012		-0.63	CJK_210215_E2;...	3120
☑	✓	(K)AHDELHSVEEIIGSNQR(D)	99%	12.8	25.0	12.8	2			56270	645.65	724	1,933.93	740	3	0	1.0		1.3	CJK_210215_E2;...	3110
☑	✓	(K)AHDELHSVEEIIGSNQR(D)	96%	7.6	25.0	7.6	2			263200	484.24	724	1,932.93	740	4	0	0.0020		1.0	CJK_210215_E2;...	3130
☑	✓	(K)AHDELHSVEEIIGSNQR(D)	95%	10.6	25.0	10.6	2			57770	645.65	724	1,933.93	740	3	0	1.00		-3.4	CJK_210215_E2;...	3140
☑	✓	(K)AHDELHSVEEIIGSNQRDEFK(G)	100%	42.7	25.0	42.7	2			667100	614.05	724	2,452.16	744	4	0	-0.00045		-0.19	CJK_210215_E2;...	3470
☑	✓	(K)AHDELHSVEEIIGSNQRDEFK(G)	99%	10.2	25.0	10.2	2			138000	614.30	724	2,453.16	744	4	0	1.0		-0.82	CJK_210215_E2;...	3460
☑	✓	(K)SSDLDKLYLSDFSFDR(L)	100%	63.5	25.0	63.5	2			2584000	636.64	878	1,906.89	893	3	0	-0.00062		-0.33	CJK_210215_E2;...	5070
☑	✓	(K)SSDLDKLYLSDFSFDR(L)	100%	68.1	25.0	68.1	2			212200	636.64	878	1,906.90	893	3	0	0.00076		0.40	CJK_210215_E2;...	5100
☑	✓	(K)SSDLDKLYLSDFSFDR(L)	100%	74.3	25.0	74.3	2			510800	636.64	878	1,906.90	893	3	0	0.00019		0.10	CJK_210215_E2;...	5090
☑	✓	(K)SSDLDKLYLSDFSFDR(L)	100%	29.1	25.0	29.1	2			102800	636.97	878	1,907.90	893	3	0	1.0		-1.3	CJK_210215_E2;...	5120
☑	✓	(K)SSDLDKLYLSDFSFDR(L)	100%	23.1	25.0	23.1	2			74890	636.97	878	1,907.90	893	3	0	1.0		-1.3	CJK_210215_E2;...	5140
☑	✓	(K)LYLSDFSFDR(L)	100%	54.0	25.0	54.0	2			1004000	631.81	884	1,261.60	893	2	0	0.000040		0.032	CJK_210215_E2;...	4870
☑	✓	(K)LYLSDFSFDR(L)	100%	22.4	25.0	22.4	2			165200	631.81	884	1,261.60	893	2	0	0.0014		1.1	CJK_210215_E2;...	4890
☑	✓	(R)LLVEKVDDNLVLR(S)	100%	49.7	25.0	49.7	2			552100	509.30	894	1,524.89	906	3	0	0.00071		0.47	CJK_210215_E2;...	3650
☑	✓	(R)LLVEKVDDNLVLR(S)	100%	57.5	25.0	57.5	2			292400	509.30	894	1,524.89	906	3	0	0.0025		1.6	CJK_210215_E2;...	3670
☑	✓	(R)LLVEKVDDNLVLR(S)	100%	68.8	25.0	68.8	2			356300	763.45	894	1,524.89	906	2	0	-0.0022		-1.4	CJK_210215_E2;...	3640
☑	✓	(R)LLVEKVDDNLVLR(S)	100%	71.3	25.0	71.3	2			2148000	509.30	894	1,524.89	906	3	0	0.0010		0.67	CJK_210215_E2;...	3640
☑	✓	(K)VDDNLVLR(S)	99%	80.8	25.0	80.8	2			234200	472.26	899	942.51	906	2	0	-0.0018		-1.9	CJK_210215_E2;...	2310
☑	✓	(K)VDDNLVLR(S)	99%	81.9	25.0	81.2	2			1411000	472.26	899	942.51	906	2	0	0.00015		0.15	CJK_210215_E2;...	2320
☑	✓	(R)SNESSHNNGVLTIKDWFK(E)	100%	34.1	25.0	34.1	2			622300	520.01	907	2,075.99	924	4	0	0.99		-8.2	CJK_210215_E2;...	4120
☑	✓	(R)SNESSHNNGVLTIKDWFK(E)	100%	30.1	25.0	30.1	2			548600	520.01	907	2,075.99	924	4	0	0.99		-8.4	CJK_210215_E2;...	4110
☑	✓	(R)SNESSHNNGVLTIKDWFK(E)	100%	24.8	25.0	24.8	2			160800	693.00	907	2,075.99	924	3	0	0.98		-8.9	CJK_210215_E2;...	3960
☑	✓	(R)SNESSHNNGVLTIKDWFK(E)	100%	16.7	25.0	16.7	2			874100	519.76	907	2,075.01	924	4	0	0.0015		0.74	CJK_210215_E2;...	3720
☑	✓	(R)SNESSHNNGVLTIKDWFK(E)	100%	18.1	25.0	18.1	2			555600	693.01	907	2,075.99	924	3	0	0.99		-7.7	CJK_210215_E2;...	4110
☑	✓	(R)KLTAENLGTYFK(N)	100%	45.1	25.0	45.1	2			5196000	462.25	943	1,383.74	954	3	0	-0.00024		-0.17	CJK_210215_E2;...	3040
☑	✓	(R)KLTAENLGTYFK(N)	100%	40.5	25.0	40.5	2			196800	692.88	943	1,383.74	954	2	0	-0.00032		-0.23	CJK_210215_E2;...	3030
☑	✓	(R)KLTAENLGTYFK(N)	100%	38.9	25.0	38.9	2			1262000	462.25	943	1,383.74	954	3	0	-0.00072		-0.52	CJK_210215_E2;...	3030
☑	✓	(R)KLTAENLGTYFK(N)	100%	38.7	25.0	38.7	2			1712000	462.25	943	1,383.74	954	3	0	-0.00012		-0.088	CJK_210215_E2;...	3060
☑	✓	(R)KLTAENLGTYFK(N)	100%	33.9	25.0	33.9	2			910500	462.25	943	1,383.74	954	3	0	-0.00039		-0.29	CJK_210215_E2;...	3070
☑	✓	(R)KLTAENLGTYFK(N)	100%	101.1	25.0	101.1	2			990000	692.88	943	1,383.74	954	2	0	-0.0014		-1.0	CJK_210215_E2;...	3040
☑	✓	(R)KLTAENLGTYFK(N)	100%	19.5	25.0	19.5	2			272600	692.88	943	1,383.74	954	2	0	-0.0013		-0.97	CJK_210215_E2;...	3060
☑	✓	(K)LTAENLGTYFK(N)	100%	45.4	25.0	45.4	2			422200	628.83	944	1,255.64	954	2	0	-0.0023		-1.8	CJK_210215_E2;...	3800
☑	✓	(K)LTAENLGTYFK(N)	100%	81.2	25.0	81.2	2			1124000	628.83	944	1,255.64	954	2	0	-0.0014		-1.1	CJK_210215_E2;...	3820
☑	✓	(K)ADNLLNYATK(E)	100%	51.9	25.0	51.9	2			2281000	561.79	959	1,121.57	968	2	0	0.00011		0.094	CJK_210215_E2;...	2740
☑	✓	(K)ADNLLNYATK(E)	100%	54.5	25.0	54.5	2			387700	561.79	959	1,121.57	968	2	0	0.00015		0.14	CJK_210215_E2;...	2750
☑	✓	(K)EDQNESNLSSLKTELSK(I)	99%	14.4	25.0	14.4	2			55860	641.32	969	1,920.93	985	3	0	0.0027		1.4	CJK_210215_E2;...	2660
☑	✓	(K)IITNAGNFGVAK(Q)	100%	56.0	25.0	52.7	2			263600	602.84	986	1,203.66	997	2	0	-0.00033		-0.27	CJK_210215_E2;...	2440
☑	✓	(K)IITNAGNFGVAK(Q)	100%	69.4	25.0	66.5	2			5477000	602.84	986	1,203.66	997	2	0	-0.000...		-0.041	CJK_210215_E2;...	2410
☑	✓	(K)IITNAGNFGVAK(Q)	100%	34.1	25.0	34.1	2			198700	602.84	986	1,203.66	997	2	0	-0.00077		-0.64	CJK_210215_E2;...	2390
☑	✓	(K)IITNAGNFGVAK(Q)	100%	79.6	25.0	76.4	2			795000	602.84	986	1,203.66	997	2	0	-0.00058		-0.48	CJK_210215_E2;...	2420

Fig. S7. The peptide report of ApxIIIA detected by LC-MS/MS showed that no post-transcriptional acetylation was found.

Table S1. *A. pleuropneumoniae* strains used in this study.

Strain	Description	Source
MIDG2331	Serovar 8 clinical isolate, genome accession number: NZ_LN908249.1	[1]
MIDG2331Δ*nlpI*	*nlpI* gene was replaced in the genome of MIDG2331 with a trimethoprim resistance cassette	[2]
MIDG2331Δ*palA*	*palA* gene was replaced in the genome of MIDG2331with a chloramphenicol resistance cassette	This study
MIDG2331Δ*nlpI*Δ*palA*	*palA* gene was replaced in the genome of MIDG2331Δ*nlpI* with a chloramphenicol resistance cassette	This study

Table S2. Primers used for construction of *A. pleuropneumoniae* mutants.

Primers	Sequence (5’-3’)
up-F	**ATCTTCTAGAAAGAT**CCGGACGGTTCTCGTATT
up-R	**TGCCGACCGCTTGTA**GTGCCGCAATCATCAATACT
Cm-F	**TGATGATTGCGGCAC**TACAAGCGGTCGGCAATAG
Cm-R	**AATACTGCGCGACGG**AACAAGCGGTTTCAACTAACG
down-F	**TTGAAACCGCTTGTT**CCGTCGCGCAGTATTAGAA
down-R	**GTTTTTCAGCAAGAT**AAGACGCAAGCAAAGATAAGC

Bold letters indicate 15bp overlaps required in in-fusion cloning.

Table S3. Primers used for qPCR detecting expression of *A. pleuropneumoniae* genes.

Primers	Sequence (5'-3')	Length of amplicon
pgaA-F	ATCAATGCGTCAAGCGATGC	193bp
pgaA-R	CTAACACGGCAACCGCTTTT	
cpxR-F	ACTGACGGAACTGTTAGCCG	218bp
cpxR-R	TTTCATCGTCTCTGGCGCTT	
dspB-F	GGCGGCACTTTTCTGCATTT	214bp
dspB-R	GGCTATCGACTTCAGGCACA	
cpxA-F	TCTCTCGTCCGGTAAAAGCG	213bp
cpxA-R	CAAGCGGCGTTTTCAGTTCA	
16sRNA-F	GATGACCAGCCACACTGGAA	210bp
16sRNA-R	GGAGTTAGCCGGTGCTTCTT	

Table S4. Primers used for qPCR detecting expression of genes in PAMs.

Gene name	Sequence (5' to 3')	Sequence (5' to 3')	Amplicon length
ACTB (6)	F: CTACGTCGCCCTGGACTTC	R: GCAGCTCGTAGCTCTTCTCC	76
ARG1 (202)	F: AATTGGCAAGGTGATGGAAG	R: TCCAGTCCATCCACATCAAA	90
B2M (7)	F: TGAAGCACGTGACTCTCGAT	R: CTCTGTGATGCCGGTTAGTG	70
CCL17 (205)	F: GGGTGGTACCAGACCTCAGA	R: GTCCTTGGGGTCAGAACAGA	90
CCL17 (766)	F: TCTGTTCTGACCCCAAGGAC	R: GGTCCTGTGGCTTCATGTTT	75
CCL19 (908)	F: CTGGACTTCTCCTGCTCTGG	R: AAAGGCTCGAACCAGATTCC	97
CCL19 (909)	F: CTGCTGCCTGTCTGTGACTC	R: AATGAGCAGGTAGCGAAAGG	90
CCL2 (293)	F:CTTCTGCACCCAGGTCCTT	R: CGCTGCATCGAGATCTTCTT	93
CCL20(995)	F: CTGCAGCAAGTCAGAAGCAG	R: GCTGTGTGAAGCCCATGATA	95
CCL20(996)	F: TATCATGGGCTTCACACAGC	R: TCTGCACACACGGCTAACTT	93
CCL22 (767)	F: CCCTGCGTGTGGTGAAGTAT	R: ATCTCTCGGTCCCTCAAGGT	88
CCL22 (768)	F: ACCTTGAGGGACCGAGAGAT	R: TCCAGCCTCTGGAGAATCTT	71
CCL26 (769)	F: CTGCTTCCAATACAGCCACA	R: AGCAGCTGTTCCTGGTGAAT	74
CCL3(236)	F: CTCTGCAGCCAGGTCTTCTC	R: CTACGAATTTGCGAGGAAGC	97
CCL5 (121)	F:CTCCATGGCAGCAGTCGT	R: AAGGCTTCCTCCATCCTAGC	121
CCL5 (611)	F:CTCCATGGCAGCAGTCGT	R: AAGGCTTCCTCCATCCTAGC	121
CCL8 (1065)	F: TTCTGTGTCTGCTGCTCACC	R: AGGGGATCTTTCCATTGACC	114
CCL8 (1066)	F: GCCAGATTCAGTCTCCATCC	R: AGGGGATCTTTCCATTGACC	62
CCR4 (906)	F: ACACCTGGACTACGCCATTC	R: TTTCTCCCCCAGGAAGAAGT	91
CCR4 (907)	F: GGACCCCTTACAATGTGGTG	R: GAATGGCGTAGTCCAGGTGT	96
CCR7(607)	F: TCCACGTCTGCAAACTCATC	R: GTCGATGCTGATGCAGAGAA	83
CD101(572)	F: ATTGAAACTCAGGCCCACAG	R: CCTGGCTGTGTTCTGTAGCA	80
CD14 (614)	F: AAGCTCACCGTGCTTGATCT	R: CCTTCCAGGGTCAGGTCAT	92
CD163 (150)	F:CACATGTGCCAACAAAATAAGAC	R: CACCACCTGAGCATCTTCAA	130
CD1A (556)	F: TAGGAGGCCAGGACATCATC	R: GGCACAATCACTGCCAATAA	76
CD206 (176)	F: GGCAATGGGTGGACAACAG	R: CACAGATCCAGGCGTTTTCT	144
CD209(586)	F: CGGAGCAGAAATTCCTGAAG	R: CATTGCCAGGAACCTTCATT	94
CD40 (9)	F: TGAGAGCCCTGGTGGTTATC	R: GCTCCTTGGTCACCTTTCTG	90
CD5L 1045	F: GCTGCTCTTCTGGGATTTTG	R: TCTCCTCCTACCAGCCTCAA	100
CD69 (267)	F: CCAAAGCTTCTGCTCCAAAC	R: GTGTTCAGCTCTCCCCACAT	100
CD69 (268)	F: CCTTGCATCCGAATAGAGGA	R: CATCTGGGCATGAAGAAACA	102
CD80 (1063)	F: CGCACCTTCACTGATGTCAC	R: CACAGGTGTAGGTGCCATTG	82
CD80 (1064)	F: TTTCAATGTGACAGGCAACC	R: TGATTAGCAGAAGAGGTTTCTCG	177
CD86(560)	F: CATCGTCTGTGTCCTGCAAC	R: CACAGGTGGCTTTGCATCTA	82
CXCL10 (111)	F:CCCACATGTTGAGATCATTGC	R: GCTTCTCTCTGTGTTCGAGGA	141
CXCL10(994)	F: TTCGCTGTACCTGCATCAAG	R: CAACATGTGGGCAAGATTGA	99

CXCL9(793)	F: AGCAGTGTTGCCTTGCTTTT	R: ATGCAGGAACAACGTCCATT	92
CXCL9(794)	F: TTAAACAATTTGCCCCAAGC	R: TGTTTGATCCCCATTCTTCA	74
CXCR4 (574)	F: ACGGGTTCCGTATATTCACTTC	R: GGAAACAGGGTTCCTTTATGG	87
GAPDH (20)	F: ACCCAGAAGACTGTGGATGG	R: AAGCAGGGATGATGTTCTGG	79
HPRTI (25)	F: ACACTGGCAAAACAATGCAA	R: TGCAACCTTGACCATCTTTG	71
IFNG (50)	F:CCATTCAAAGGAGCATGGAT	R: TTCAGTTTCCCAGAGCTACCA	76
IFNG (51)	F: GAATTGGAAAGAGGAGAGTGACA	R: TCACATCCATGCTCCTTTGA	116
IL10 (133)	F: TACAACAGGGGCTTGCTCTT	R: GCCAGGAAGATCAGGCAATA	110
IL13 (109)	F: CCAAGCGAGCAAGTTCCTG	R: AACTACCCGTGGCGAAAAAT	110
IL13 (297)	F: GAAGACACCCCTATGCAACG	R: GCAGTCGGAGATGTTGATGA	100
IL13RA1 (914)	F: CAATGCGGGAAAAATCAGAC	R: TATGCGGAGGATCAGGTTTC	74
IL13RA1 (915)	F: TCCCTCCAATTCCTGATCCT	R: TCCAGTGCAGGGTATCATCA	75
IL13RA2 (209)	F: TGAAAGCTGGAAGACGATCA	R: GCCCTGGCAGAAGTGTATGT	101
IL15(292)	F: CGTCATTTTGCAAGAGTCCA	R: TGGACGATAAACTGCTGTTTGC	86
IL18 (234)	F: CAATTGCATCAGCTTTGTGG	R: TCCAGGTCCTCATCGTTTTC	78
IL1A (157)	F: TGTGCTAAATAACCTGGATGAGG	R: GGTTCGTCTTCGTTTTGAGC	135
IL1B (233)	F: TCTCTCACCCCTTCTCCTCA	R: GACCCTAGTGTGCCATGGTT	60
IL23 (1350)	F: CAACAGTCAGTCCTGCTTGC	R: GCTCCCCTGTGAAAATGTCT	86
IL23 (1351)	F: GGACATGTGGATCTACCAAGAGA	R: GAGTCCCTGAGGATCACAGC	96
IL4 (108)	F: GCAAACATGACCTGTTCTGTG	R: GCTTCAACACTTTGAGTATTTCTCC	105
IL4R (912)	F: CTGGCACTCTTGGGTGTCTC	R: TGGAGCTCTGAACATTGCTG	79
IL5 (771)	F: GGGGAAAGATGGAGAGTAACG	R: CTTTCCATTGTCCACTCGGTA	83
IL6 (232)	F:CCTCTCCGGACAAAACTGAA	R: TCTGCCAGTACCTCCTTGCT	118
IL8 (37)	F: GAAGAGAACTGAGAAGCAACAACA	R: TTGTGTTGGCATCTTTACTGAGA	99
LBP (83)	F: ATTCAATGTGGAGCTGTT	R: GGAAGCCTTCTGCCAACT	92
MHC1 (1511)	F: GGGTCCCCACTCCCTAAG	R: ACGTAGCCGACTTCGATGA	84
MHC1 (1512)	F: GGGTCCCCACTCCCTAAG	R: CGTCCACGTAGCCGACTT	89
MHC2 (1513)	F: CGCCTCGACACAGAATCTC	R: AGCAGAATGAGGATGGCTTG	71
MYD88 (179)	F: CCAGACTAAGTTTGCACTCAGC	R: AGGATGCTGGGGAACTCTTT	99
NOD-1 (366)	F: CAGTGGGGTGAAGGTGCTAT	R: TACCTGGCTCCGACATCAGT	99
NOD-2 (145)	F: GAAAGTCCTGAAGCTGTCCAAC	R: CCAGACTTCCAGGATGGTGT	97
NOD-2 (609)	F: AGCTCGTGGAACATGCTCTT	R: CGTCGGTCAATTTGTTGTTG	72
RPL13A (58)	F: ATTGTGGCCAAGCAGGTACT	R: AATTGCCAGAAATGTTGATGC	76
STAT6 (254)	F: TCCCAGATGTATCCACCACA	R: ATCTGCAGGTGAGGTTCCTG	107
STAT6 (772)	F: GGCAGAAGAGAGTTGCCTGA	R: CAAGGGTGGGAACATGTCTT	82
TLR1 (1302)	F: GAATTTCTGGGGTTGAGTGC	R: GGGTCTTCTCTTTCCCCGTA	119
TLR1 (188)	F: CCTTCAAGACCTTAACACACAGAG	R: CAGATTTACTGCGGTGCTGA	100
TLR2 (1091)	F: TCCGAGAACTTTGTGAAGAGC	R: ATGGGTTCCAGCAGAATGAG	110
TLR2 (160)	F: CGGAGGTTGCATATTCCACAG	R: TGTGAAAGGGAACAGGGAAC	128

TLR3 (123)	F: ATTGTGCAAAAGATTCAAGGTG	R: TCTTCGCAAACAGAGTGCAT	130
TLR3 (161)	F: ACATCTACTGAAAGATCCATTGTGC	R: TCTTCGCAAACAGAGTGCAT	148
TLR4 (62)	F: TTTCCACAAAAGTCGGAAGG	R: CAACTTCTGCAGGACGATGA	145
TLR4(235)	F: TGGTGTCCCAGCACTTCATA	R: CAACTTCTGCAGGACGATGA	116
TLR5 (187)	F: AGTTCTGAACCTGGCCTTCA	R: TAAGCGAGCTTAGGCAGTCC	144
TLR5 (364)	F: CGCTTGGACCTATCCAAAAA	R: GATCAATGGCCTTCAAGGAA	85
TLR6 (1303)	F: CCAAATAGCTTCTTCTCTGTCCA	R: ATTTAGTAAGGTTGGCCCTTGAG	147
TLR6 (163)	F: TGGATGTTAGCTCGAATTCTTTG	R: GAACCTTGATCCTGGGAGGT	141
TLR7 (164)	F: GGAAATAGCATCAGCCAAGCTC	R: TTCCAGGTTGCGTAGCTCTT	132
TLR8 (183)	F: GCAAAGACCACCACCAACTT	R: ATCCGTCAGTCTGGGAATTG	129
TLR9 (99)	F: CCTGTTCTATGATGCCTTCGTG	R: GGTACCCAGTCTCGCTCCTC	144
TNFA (74)	F:CCCCCAGAAGGAAGAGTTTC	R: CGGGCTTATCTGAGGTTTGA	92

References

1. Bosse, J.T., et al., *Complete Genome Sequence of MIDG2331, a Genetically Tractable Serovar 8 Clinical Isolate of Actinobacillus pleuropneumoniae.* Genome Announc, 2016. **4**(1).
2. Antenucci, F., et al., *Identification and characterization of serovar-independent immunogens in Actinobacillus pleuropneumoniae.* Vet Res, 2017. **48**(1): p. 74.
